# Relationship of rotating-shift work with lifestyle and health status of hospital employees

**DOI:** 10.1371/journal.pgph.0006956

**Published:** 2026-08-19

**Authors:** Maria Antonia Alou-Soler, Aina M. Galmes-Panades, Ángel Arturo López-González, Aina M. Yañez, Miquel Bennasar-Veny

**Affiliations:** 1 Research Group on Global Health, University of the Balearic Islands, Palma, Spain; 2 Research Group on Nursing, Community & Global Health, Health Research Institute of the Balearic Islands (IdISBa), Palma, Spain; 3 Department of Nursing and Physiotherapy, University of the Balearic Islands (UIB), Palma, Spain; 4 CIBER of Physiopathology of Obesity and Nutrition (CIBEROBN), Instituto de Salud Carlos III, Madrid, Spain; 5 Physical Activity and Sport Sciences Research Group (GICAFE), Institute for Educational Research and Innovation, University of the Balearic Islands, Palma, Spain; 6 ADEMA University School, Palma, Spain; 7 Prevention of Occupational Risks in Health Services, Balearic Islands Health Service (IB-Salut), Palma, Spain; 8 Research Network on Chronicity, Primary Care, and Health Promotion (RICAPPS), Institute of Health Carlos III, Madrid, Spain; 9 Centre for Biomedical Research Network (CIBER) in Epidemiology and Public Health (CIBERESP), Madrid, Spain; PLOS: Public Library of Science, UNITED STATES OF AMERICA

## Abstract

Night shift work has been associated with adverse health effects, although findings remain inconsistent. This study aimed to analyze the association between night shift work and sleep quality, chronotype, cardiometabolic parameters, and behavioral factors among hospital workers. A cross-sectional study was conducted among 225 normoglycemic workers at Son Llàtzer University Hospital (Mallorca, Spain). Sociodemographic and occupational data were collected through a structured questionnaire. Physical activity was assessed using the International Physical Activity Questionnaire (IPAQ), adherence to Mediterranean diet with the 14-item PREDIMED tool, sleep quality with the Pittsburgh Sleep Quality Index (PSQI), and chronotype with the Morningness-Eveningness Questionnaire (MEQ). Clinical parameters, including fasting plasma glucose, lipid profile, blood pressure, body mass index (BMI), waist circumference, and cardiovascular risk, were obtained from standardized health assessments. Associations with night shift work were examined using bivariate analyses and general linear models adjusted for age, sex, BMI, and physical activity. Night shift work was not significantly associated with fasting plasma glucose, triglycerides, HDL cholesterol, sleep quality, or chronotype. Compared with day workers, night shift workers showed lower levels of total and LDL cholesterol, blood pressure, BMI, and waist circumference, lower smoking rates, lower cardiovascular risk, and higher physical activity levels. No significant differences were observed in adherence to the Mediterranean diet. After adjustment for age and sex, only the association between night shift work and physical activity remained significant. Overall, night shift work was not associated with poorer health status or increased cardiovascular risk. Instead, no consistent evidence of a less favourable lifestyle was observed among night shift workers.

## Introduction

Sleep disturbances and sleep deprivation are highly prevalent in modern societies, and these conditions are associated with many adverse outcomes, including metabolic, cardiovascular, psychiatric, neurological, and immunological disorders. These effects are largely mediated by systemic inflammation and hormonal dysregulation, and can contribute to metabolic dysfunction, such as insulin resistance, dyslipidemia, obesity, increased cardiovascular risk (CVR), and mental health problems [1,2].

In Spain, the prevalence of symptoms of insomnia in adults ranges between 20% and 43%, depending on the diagnostic criteria, and about 13–14% of people meet the criteria for chronic insomnia disorder [3,4]. A recent population-based study in Spain found more than one-third of adults reported poor sleep quality [5]. Shift work, particularly work at night, is a major occupational determinant of poor sleep quality because it disrupts the natural sleep-wake cycle and circadian rhythm [6,7], and these changes affect hormonal regulation, most notably the secretion of insulin and cortisol, and can lead to impaired glycemic control [8,9]. Despite identification of these associations, it is still unclear whether rotating-shift work is independently associated with poorer glycemic control [10,11].

A rotating-shift schedule can disrupt the endogenous circadian rhythm and increase the risk of sleep disorders [6], and only about 35% of rotating-shift workers achieve partial circadian adaptation. This misalignment of the work schedule and the body’s circadian rhythm is associated with insomnia, excessive daytime sleepiness, and prolonged wakefulness, and working many consecutive night shifts can significantly impair the quality of life [6,7,12]. Nevertheless, rotating-shift work is essential in many occupations and settings, particularly in healthcare [6,7].

Long-term exposure to rotating-shift work is also associated with hypertension, endothelial dysfunction, and chronic low-grade inflammation, conditions that contribute to the development of cardiovascular disease (CVD) [13]. One of the prospective studies that was part of the Nurses’ Health Study reported that more than 10 years of exposure to a rotating-shift was associated with an increased CVR, although this association declined after 20 years of follow-up [14]. Other research reported that chronic sleep deprivation, a common consequence of rotating-shift work, was independently associated with obesity, hypertension, and increased risk of CVD [15]. A 2024 systematic review and meta-analysis concluded that individuals who had fewer than 6 h of sleep per night had a significantly higher metabolic risk and CVR than those who had 7 or 8 h of sleep per night [7].

Type 2 diabetes mellitus (T2DM), a leading cause of CVD and other chronic conditions, currently affects an estimated 462 million individuals (approximately 6.3% of the global population) and was responsible for more than 1 million deaths in 2017 [16,17]. There is evidence that links rotating-shift work to a higher risk of metabolic disorders, including insulin resistance and T2DM [8,11,18], but there are also some inconsistent findings [10].

Chronobiology researchers have identified people with different circadian preferences, or chronotypes, and classified people as ‘morning’, ‘evening’, or ‘intermediate’ types. Most individuals exhibit an intermediate chronotype, but occupational demands can lead to misalignment of the work schedule with the natural circadian inclination. This desynchronization is particularly pronounced in rotating-shift workers, and leads to fatigue, metabolic strain, and sleepiness [18,19]. Evening chronotypes are especially vulnerable, because they tend to accumulate greater sleep debt when exposed to irregular or nocturnal work schedules, and they often attempt to compensate for this disruption on non-working days [20].

Given these somewhat heterogeneous findings, further research is needed to clarify the factors that influence the association between rotating-shift work and glycemic control. Therefore, this study examined the association between rotating-shift work, clinical parameters, and CVR in hospital workers at Son Llàtzer University Hospital, Spain. This issue is of particular relevance in the healthcare sector, where shift work is widespread.

## Materials and methods

### Ethics statement

The study protocol and data collection procedures were approved by the Health Research Ethics Committees of the Balearic Islands (CEI-IB, Ref. No.: IB 5011/22 PI). In addition, the Research Committee of Son Llàtzer University Hospital reviewed and supported the study. The research was conducted according to the principles of the Declaration of Helsinki. All participants provided written informed consent.

### Study design

This cross-sectional study examined 225 normoglycemic hospital workers from Son Llàtzer University Hospital, Mallorca, Balearic Islands, Spain. Participants were recruited by convenience sampling of employees who received an occupational health assessment during the previous year, as part of this hospital’s Occupational Risk Prevention Plan. The study population consisted of physicians, nurses, midwives, nursing assistants, orderlies, administrative staff, and telephone operators.

All participants were between 18 and 70-years-old. The exclusion criteria were diagnosis of T1DM or T2DM, current use of an oral antidiabetic medication, terminal illness, pregnancy, major surgery within the previous three months, participation in another study, or missing data. The Occupational Risk Prevention Service initially contacted eligible participants by telephone. Recruitment was conducted between March and October 2023.

### Sample size

No sample size calculation was performed before participant recruitment. As a post hoc assessment, we estimated that a sample of 225 participants would provide adequate statistical power to detect clinically meaningful between-group differences in Framingham cardiovascular risk. Assuming a two-sided alpha level of 0.05, 80% power, and approximately equal group sizes, a sample of 225 participants would allow the detection of a standardized mean difference of approximately 0.37, corresponding to a small-to-moderate effect size. Considering a plausible standard deviation of 7–8 percentage points for the Framingham risk score, this translates into the ability to detect absolute differences of approximately 2.6–3.0 percentage points between groups.

### Data collection

Sociodemographic data (age, sex, cohabitation status, marital status, and education level), occupational and sleep-related variables (job category, contract type, rotating-shift status, shift duration, sleep duration, and years of rotating-shift work) were collected using a purpose-designed questionnaire. Physical activity was assessed using the validated International Physical Activity Questionnaire (IPAQ) [21], and was categorized as ‘light’, ‘moderate’, or ‘vigorous’; daily and weekly totals of exercise (min) at each intensity were calculated [22]. Adherence to the Mediterranean diet was assessed with the 14-item PREDIMED questionnaire; a score below 9 indicated ‘low adherence’ and a score of 9 or more indicated ‘high adherence’ [23]. Sleep quality was evaluated using the Pittsburgh Sleep Quality Index (PSQI); a score below 5 indicated ‘good’ and a score of 5 or more indicated ‘poor’ [24,25]. Chronotype was determined using the Spanish version of the Morningness-Eveningness Questionnaire (MEQ) [26]; a score of 41 or less indicated ‘evening type’, 42–58 indicated ‘intermediate type’, and 59 or more indicated ‘morning type’ [27]. Tobacco use was recorded as ‘current’, ‘former’, or ‘non-smoker’. Use of sleep medication was also recorded.

Cardiovascular risk (CVR) was estimated using the Framingham risk equation [28] and was classified as ‘low’, ‘intermediate’, or ‘high’. Clinical parameters, including fasting plasma glucose (FPG), total cholesterol, LDL cholesterol (LDL-c), HDL cholesterol (HDL-c), and triglycerides (TG), were also assessed during this examination. Anthropometric measurements (body mass index [BMI] and waist circumference [WC]) and blood pressure (BP) were also measured during this examination.

Rotating-shift work in this setting typically follows a forward rotation schedule consisting of a morning shift (08:00–15:00), an afternoon shift (15:00–22:00), and a night shift (22:00–08:00), followed by a post-night rest day and an additional day off. However, due to organizational adjustments and shift exchanges between workers, individual schedules may vary. On average, participants reported performing approximately 4–6 night shifts per month. Information on years of exposure to rotating-shift work was also collected using a purpose-designed questionnaire, as previously described. In contrast, day-shift work consisted of either fixed morning shifts (08:00–15:00) or predominantly daytime schedules that may include morning shifts (08:00–15:00) and afternoon shifts (15:00–22:00), depending on professional category.

In this study, circadian alignment/misalignment was defined based on the concordance between individual chronotype and work schedule. Chronotype was assessed using the Morningness–Eveningness Questionnaire (MEQ) [18–20]. Alignment was defined as morning chronotype in day-shift workers and evening chronotype in rotating-shift workers, whereas misalignment was defined as evening chronotype in day-shift workers and morning chronotype in rotating-shift workers. Participants with intermediate or undefined chronotype were excluded from this analysis to ensure a clear binary classification.

### Statistical analyses

Descriptive statistics are presented as means and standard deviations (SDs) for continuous variables, and as absolute frequencies and percentages for categorical variables. Bivariate analysis was performed to compare rotating-shift *vs.* day-shift workers, and to compare men and women (see S1 Table). To determine the significance of differences, an independent t-test was used for continuous variables, and a Chi-square test was used for categorical variables. Parametric tests were used only when the normality assumption was satisfied.

A general linear model (GLM) was used to examine the association of rotating-shift work with FPG, HDL cholesterol, TG, and blood pressure, after adjusting for age, sex, BMI, and total PA. Covariates were selected based on prior evidence and their potential role as confounders in the relationship between shift work and cardiometabolic outcomes. Estimated marginal means of PA (MET-min/day) were calculated to assess the association of rotating-shift work with PA, with stratification by age and sex.

All analyses were performed using IBM SPSS Statistics version 25 (SPSS/IBM, Chicago, IL, USA). A p-value below 0.05 was considered statistically significant.

## Results

### General characteristics of participants

A total of 240 individuals participated in the study, and 225 had valid data for the analyses (Table 1). The mean age was 42.3 years (SD: 11.2; range: 22–64), and there were 176 women (78.2%) and 49 men (21.8%). The main occupational categories were nursing staff, midwives, and residents (48.9%); physicians and medical residents (19.1%); and administrative staff (17.3%, included in the ‘Others’ category). Overall, 64% of the participants were employed under a permanent statutory contract, and the others were employed as residents (19.6%) or temporary workers (16.4%). A total of 45.3% of the participants worked a permanent day shift, and 54.7% worked a rotating night shift (henceforth, ‘night shift’). Among those with a night shift, 39.1% reported more than four night shifts per month. Most day shifts (86.2%) lasted 7–10 h, whereas 47.2% of night shifts lasted 7–10 h and 52.8% lasted 12–24 h.

**Table 1:** Bivariate analysis of the association of different variables with work schedule.

Variable	All workers	Rotating-Shift	Day- Shift	p-value
	(n=225)	(n=123)	(n=102)	
Mean ± SD/ N (%)				
***Sociodemographic and occupational variables***				
Age (years)	42.3 ± 11.2	38.0 ± 10.1	47.4 ± 10.3	<0.001*
Work duration (years)	11.9 ± 9.7	11.3 ± 9.4	12.6 ± 10.1	0.314
Sex				0.044*
Woman	176 (78.2)	90 (73.2)	86 (84.3)	
Men	49 (21.8)	33 (26.8)	16 (15.7)	
Living with others				0.382
Yes	168 (74.7)	89 (72.4)	79 (77.5)	
No	57 (25.3)	34 (27.6)	23 (22.5)	
Marital Status				0.956
In a relationship	135 (60.0)	74 (60.2)	61 (59.8)	
Single	90 (40.0)	49 (39.8)	41 (40.2)	
Education Level				0.002*
University Degree	140 (62.2)	88 (71.5)	52 (51.0)	
Non-University Degree	85 (37.8)	35 (28.5)	50 (49.0)	
Job Category				<0.001*
Nursing Staff, Midwives and Residents	110 (48.9)	72 (58.5)	38 (37.3)	
Physicians and Medical Residents	43 (19.1)	39 (31.7)	4 (3.9)	
Orderlies	17 (7.6)	11 (8.9)	6 (5.9)	
Others	55 (24.4)	1 (0.8)	54 (52.9)	
Contract Type				<0.001*
Permanent	144 (64.0)	65 (52.8)	79 (77.5)	
Resident	44 (19.6)	36 (29.3)	8 (7.8)	
Temporary	37 (16.4)	22 (17.9)	15 (14.7)	
***Lifestyle variables***				
Adherence to Mediterranean Diet	8.7 ± 1.9	8.6 ± 2.0	8.8 ± 1.7	0.563
Light Physical Activity (min/day)	1.4 ± 6.0	1.3 ± 6.6	1.6 ± 5.3	0.715
Moderate Physical Activity (min/day)	44.1 ± 63.6	52.8 ± 67.0	33.6 ± 57.9	0.024*
Vigorous Physical Activity (min/day)	20.3 ± 40.9	27.0 ± 48.9	12.1 ± 26.4	0.006*
Total Physical Activity (min/day)	65.8 ± 81.3	81.1 ± 88.4	47.3 ± 67.8	0.002*
Smoking				0.045*
Non-smoker	139 (61.8)	85 (69.1)	54 (52.9)	
Smoker	35 (15.6)	15 (12.2)	20 (19.6)	
Former Smoker	51 (22.7)	23 (18.7)	28 (27.5)	

An asterisk indicates a significant difference (p < 0.05) between groups

^a^Values are indicated as mean ± SD for continuous variables and n (%) for categorical variables. Independent t-test and χ^2^ tests were used to compare continuous and categorical variables, respectively.

^b^The ‘Others’ category includes administrative assistant, administrative staff, social worker, psychologist, clinical psychology resident, pharmacist, pathology technician, biologist, telephone operator, management technician, and X-ray technician.

### Analysis of chronotype, sex, education, and employment status

Analysis of chronotype in the rotating-shift workers indicated that 32.5% had morningness, 8.9% had eveningness, and 58.5% were intermediate (Table 2). This distribution was not significantly different from that of the day-shift workers (p = 0.705). However, rotating-shift workers were majority women (73.2% *vs.* 26.8%; p = 0.044), younger (38.03 ± 10.06 *vs.* 47.4 ± 10.3 years; p < 0.001), have a university degree (71.5% *vs.* 51.0%; p = 0.002), and be employed as nurses, midwives, or nursing residents (58.5% *vs.* 37.3%; p < 0.001). In addition, 52.8% of rotating-shift workers had a permanent contract, and 77.5% of day shift workers had a permanent contract (p < 0.001) (Table 1).

**Table 2:** Bivariate analysis of the association of different variables with work schedule.

Variable	All workers	Rotating-Shift	Day- Shift	p-value
	(n=225)	(n=123)	(n=102)	
Mean ± SD/ N (%)				
***Clinical variables***				
Fasting Plasma Glucose (mg/dL)	88.8 ± 12.2	87.4 ± 12.8	90.57 ± 11.2	0.051
Total Cholesterol (mg/dL)	187.4 ± 36.6	182.3 ± 35.0	193.5 ± 37.6	0.022*
HDL Cholesterol (mg/dL)	59.4 ± 13.3	59.9 ± 12.9	58.8 ± 13.9	0.513
LDL Cholesterol (mg/dL)	113.5 ± 32.1	107.7 ± 32.1	120.5 ± 30.9	0.003*
Triglycerides (mg/dL)	77.7 ± 42.7	75.2 ± 42.8	80.7 ± 42.7	0.34
Systolic Blood Pressure (mmHg)	122.2 ± 14.8	120.3 ± 13.1	124.5 ± 16.4	0.033*
Diastolic Blood Pressure (mmHg)	75.4 ± 9.6	73.9 ± 8.4	77.1 ± 10.7	0.013*
***Anthropometric variables***				
BMI (kg/m²)	24.5 ± 5.0	23.9 ± 4.1	25.3 ± 5.9	0.034*
Waist Circumference (cm)	79.8 ± 12.6	78.0 ± 11.3	82.0 ± 13.8	0.016*
***Cardiovascular Risk (Framingham)***				
Cardiovascular Risk				0.002*
Low	205 (91.1)	119 (96.7)	86 (84.2)	
Intermediate - High	20 (8.9)	4 (3.3)	16 (15.7)	
***Sleep variables***				
Chronotype				0.705
Eveningness	23 (10.2)	11 (8.9)	12 (11.8)	
Indeterminate	127 (56.4)	72 (58.5)	55 (53.9)	
Morningness	75 (33.3)	40 (32.5)	35 (34.3)	
Sleep Quality				0.687
Good	101 (44.9)	57 (46.3)	44 (43.1)	0.077
Bad	124 (55.1)	66 (53.7)	58 (56.9)	
Sleep Duration				
≥7 h	52 (23.1)	34 (27.6)	18 (17.6)	
<7 h	173 (76.9)	89 (72.4)	84 (82.4)	
Use of Sleep Medication				0.027*
Yes	43 (19.1)	17 (13.8)	26 (25.5)	
No	182 (80.9)	106 (86.2)	76 (74.5)	

Abbreviations: HDL: High-Density Lipoprotein; LDL: Low-Density Lipoprotein; BMI: Body Mass Index.

An asterisk indicates a significant difference (p < 0.05) between groups.

a Values are indicated as mean ± SD for continuous variables and n (%) for categorical variables. Independent t-test and χ^2^ tests were used to compare continuous and categorical variables, respectively. Cardiovascular risk was recoded into a binary variable (low vs. moderate-high risk) and analyzed using Fisher’s exact test.

### Analysis of clinical parameters and characteristics of sleep

Analysis of the clinical and anthropometric variables (Table 2) indicated that the two groups had no significant differences in FPG (87.39 ± 12.81 *vs.* 90.57 ± 11.17; p = 0.051), but the rotating-shift workers had lower levels of total cholesterol (182.30 ± 35.04 *vs.* 193.50 ± 37.60; p = 0.022), LDL-c (107.65 ± 32.12 *vs.* 120.51 ± 30.88; p = 0.003), systolic blood pressure (120.31 ± 13.08 *vs.* 124.52 ± 16.39; p = 0.033), diastolic BP (73.94 ± 8.41 *vs.* 77.13 ± 10.69; p = 0.013), BMI (23.85 ± 4.05 *vs.* 25.26 ± 5.86; p = 0.034) and WC (77.95 ± 11.26 *vs.* 82.00 ± 13.79; p = 0.016). The two groups had no significant differences in sleep quality or sleep duration. Overall, 55.1% of the participants reported poor sleep quality, and 76.9% slept fewer than 7 h per night. Day-shift workers were more likely to use sleep medication (25.5% *vs.* 13.8%; p = 0.027).

### Association of clinical variables with rotating-shift work in a GLM

Table 3 shows the *β* coefficients and 95% CIs for the associations between rotating-shift work and four clinical parameters after application of a GLM that adjusted for age, sex, BMI, and total physical activity. These results show that rotating-shift work was not significantly associated with FPG, systolic BP, TG, or HDL-c after adjustment for age, sex, BMI, and total physical activity. However, as expected, BMI had significant positive associations with FPG (β = 0.349; p = 0.042), systolic BP (β = 0.82; p < 0.001), and TG (β = 2.14; p < 0.001), and a negative association with HDL-c (β=−0.80; p < 0.001) (see S2 Table). Physical activity was not significantly associated with any of the clinical parameters (see S2 Table). Additional analyses stratified by age tertiles (22–37, 38–48, and 49–64 years) showed no significant associations (see S3 Table). General linear models were used to assess the interaction between sex and rotating-shift work for clinical variables, including fasting plasma glucose, systolic blood pressure, triglycerides, and HDL cholesterol (see S4 Table).

**Table 3:** General linear model analysis of the association of rotating-shift workers with four clinical variables.

Dependent variable	β	95% CI	p-value
Fasting Plasma Glucose	−1.068	−4.572, 2.436	0.549
Systolic Blood Pressure	−1.933	−5.814, 1.948	0.327
Triglycerides	0.598	−10.930, 12.125	0.919
HDL Cholesterol	1.316	−2.222, 4.855	0.464

Abbreviations: HDL: High-Density Lipoprotein

^a^Values are presented as regression coefficients (β), 95% confidence intervals (CI), and p-values, and each model was adjusted for age, sex, Body Mass Index (BMI), and total physical activity.

### Analysis of CVR and lifestyle factors

Analysis of CVR using the Framingham equation and without adjustment for confounding (Table 2) indicated that rotating-shift workers had lower prevalences of ‘intermediate-high risk’ (3.3% *vs.*15.7%) and a greater prevalence of ‘low risk’ (96.7% *vs.* 84.2%) (p = 0.002). However, this association was not significant in a logistic regression model after adjustment for age (see S5 Table).

Overall, 54.2% of the participants had good adherence to the Mediterranean diet, with no significant differences between the two groups (Table 1). Rotating-shift workers reported significantly higher levels of moderate (p = 0.024), vigorous (p = 0.006), and total physical activity (p = 0.002). The prevalence of smoking was greater in the day-shift workers (19.6% *vs.* 12.2%; p = 0.045).

**Fig 1:**
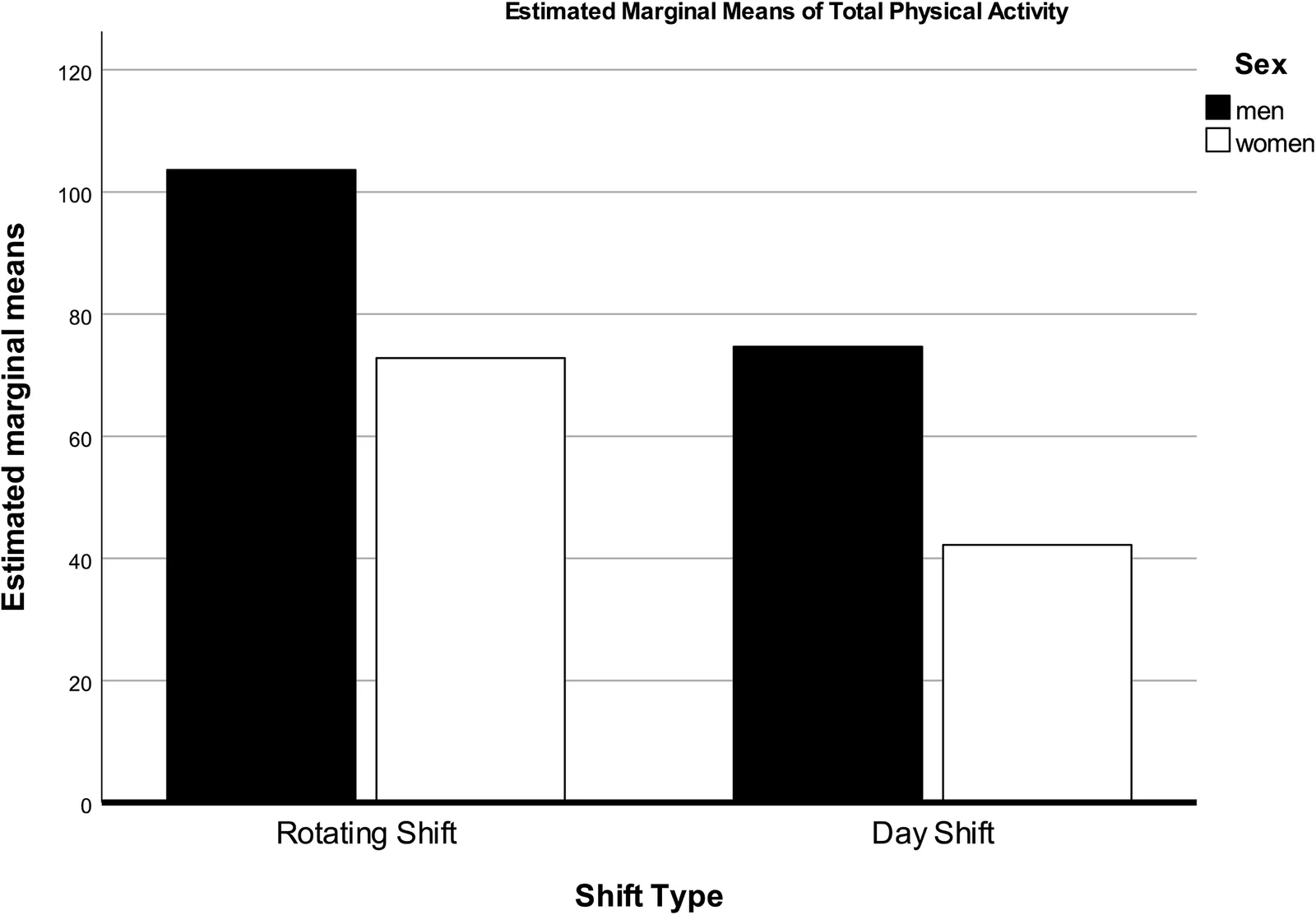
Estimated Marginal Means of Total Physical Activity.

We also used a GLM to identify the effect of sex and work shift on total daily physical activity (MET-min/day) after adjustment for age and sex (Fig 1). Men had greater total physical activity than women (p = 0.005) and rotating-shift workers had greater total physical activity than day-shift workers (p = 0.002), but age was not significantly associated with physical activity (p = 0.916). Both men and women in the rotating-shift group had more daily physical activity than their day-working counterparts (see S6 Table).

Differences in mean time of physical activity (MET-min/day) of day-shift workers and rotating-shift workers by sex, with adjustment for age. There were significant differences between rotating-shift and day-shift workers (p = 0.002) and between men and women (p = 0.005).

### Exploratory analysis of circadian alignment

We then performed an exploratory analysis to assess the association between circadian alignment (aligned *vs.* misaligned) and sociodemographic, occupational, clinical, and lifestyle variables using independent t-test and Chi-square tests (see S7 Table). Analyses were restricted to participants with a defined circadian alignment status (n = 98), excluding those with intermediate chronotype. There were no statistically significant associations of sociodemographic and occupational characteristics (age and sex), clinical parameters (FPG, total cholesterol, HDL-c, LDL-c, TG, BMI, and waist circumference), lifestyle factors (dietary habits, physical activity, and tobacco use), CVR, or sleep quality with circadian misalignment.

There were significant differences in circadian misalignment between rotating-shift workers (76.9%) compared to day-shift workers (23.1%) (p < 0.001); that is, circadian misalignment primarily affected rotating-shift workers performing night shifts despite having a morningness chronotype.

Furthermore, workers with circadian misalignment reported significantly higher levels of vigorous physical activity (30.95 ± 59.58 min/day) compared with those with circadian alignment (10.75 ± 18.71 min/day; p = 0.046) (see S7 Table).

## Discussion

This cross-sectional study of hospital workers demonstrated that rotating-shift work was not associated with unfavorable clinical and anthropomorphic parameters (FPG, total cholesterol, HDL-c, LDL-c, TG, BP, BMI, and waist circumference), lifestyle factors (dietary habits, light and moderate physical activity, tobacco use, chronotype, and sleep quality), or increased CVR based on the Framingham score. However, there was a significant and independent association of rotating-shift work with higher levels of vigorous physical activity.

Our findings are inconsistent with the hypotheses that rotating-shift work is associated with poor sleep quality, circadian misalignment, and an unhealthy cardiometabolic profile. Instead, we found no significant difference in the FPG levels between night and day workers after adjusting for sociodemographic and behavioral factors, in agreement with previous research [10]. In contrast, other studies reported a positive association of rotating-shift work with an increased level of FPG [11], although this association may be moderated by individual characteristics, such as age, physical activity, and dietary habits [9]. A recent meta-analysis of shift workers identified a higher risk of T2DM in women and individuals who were overweight or obese, but not in men or individuals with a BMI below 30 [11]. This suggests that the susceptibility to glycemic alterations in rotating-shift workers varies among different populations [9,10] and is influenced by other factors. For example, the relatively young age of our participants (42.3 ± 11.2 years) and their short cumulative exposure to night work may have attenuated the potentially adverse effects of night work.

There is conflicting evidence regarding the cardiometabolic risk of rotating-shift workers. In the present study, rotating-shift workers had a lower BMI, WC, total and LDL-c, and systolic and diastolic BP. These findings are consistent with a previous meta-analysis of 74,651 nurses, which found no increased risk of obesity among shift workers compared with day workers, even in female-only subgroups [29]. This emphasizes that the characteristics of certain populations may modulate the relationship between rotating-shift work and adiposity. Compensatory behaviors, such as higher physical activity or more structured eating patterns (as observed in our sample of night workers), may contribute to an attenuated cardiometabolic risk. Similarly, other studies have reported a lower BMI and WC in rotating-shift workers, anthropometric variables that are associated with reduced visceral adipose tissue [30] and lower cardiometabolic risk [31]. By contrast, a systematic review identified poorer lipid profiles (lower HDL-c and greater TG and LDL-c) in permanent rotating-shift workers, particularly in older populations (mean age: 42.0 to 46.4 years) [32]. Another study reported slightly greater BMI, WC, and adiposity among rotating-shift workers, and attributed this to behavioral and physiological factors, such as nighttime snacking, dysregulation of hormones (leptin and ghrelin), and sedentary behavior [33].

Although we did not directly assess metabolic flexibility in our study population, previous research highlighted the importance of the capacity of an individual to adapt metabolic processes under physiologically adverse or non-standard conditions. This adaptive capacity includes the ability to efficiently switch between energy substrates (glucose and lipids) during periods of circadian disruption, irregular eating patterns, or sleep restriction, and may be a protective mechanism [34,35]. Advanced age can impair metabolic flexibility and increase the susceptibility to circadian misalignment [36,37]. Given that our study population consisted of middle-aged adults, our findings may reflect their greater metabolic flexibility and enhanced capacity to adapt to altered sleep cycles. Unmeasured behavioral and organizational variables during working hours may have also influenced our results.

In addition, the rotating-shift workers in our population had a decreased CVR; only 2.4% of night workers had ‘high risk’, but 4.9% of day workers had ‘high risk’. In contrast, other studies reported associations between night work and endothelial dysfunction, hypertension, and elevated rates of cardiovascular events [13,15].

We observed no significant differences in adherence to the Mediterranean diet between rotating-shift and day-shift workers. In contrast, a previous study reported poorer diet quality and disrupted eating patterns in night workers [33], although another study reported healthier food choices in workers with rotating shifts [38]. These discrepancies may reflect compensatory dietary strategies or a moderating influence of organizational or food environment. Further studies should examine the impact of meal timing and dietary content in shift workers, ideally by considering multiple objective nutritional biomarkers.

We found no significant association between rotating-shift work and quality of sleep. This contrasts with certain previous studies that reported a high prevalence of poor quality sleep in healthcare workers who had rotating shifts [37,39] and an association of prolonged or irregular shifts with adverse health outcomes, including increased daytime sleepiness [40]. However, our findings regarding sleep quality are consistent with a previous study [41], suggesting that workplace structure may exert less influence on sleep quality than previously assumed. Our findings support the hypothesis that behavioral adaptation may mitigate the negative effects of circadian misalignment [42]. One possible explanation for the absence of sleep disturbances in our rotating-shift workers may be that their work schedules were rotated. Thus, our rotating-shift workers may have adopted compensatory behaviors, including higher engagement in physical activity and healthier eating patterns, as reflected in some of the variables we recorded. These strategies could help to preserve circadian alignment and reduce sleep disruptions. Prior research suggested that chronotype-related health effects are often mediated by psychological, behavioral, and contextual factors [43]. Interestingly, the rotating-shift workers in our population also reported lower rates of smoking. Although some studies reported greater smoking by males who were night workers [15], and other studies reported no significant associations of smoking with work shift or sex [44].

Our analysis of physical activity indicated that males had higher levels of vigorous and total physical activity than females, and that rotating-shift workers engaged in significantly greater total daily physical activity than day-shift workers, independent of age and sex. These findings are consistent with previous studies, which found that rotating-shift workers tended to engage in more moderate physical activity [45] and that, despite being more sedentary during work hours, they may compensate with increased physical and recreational activities during their free time [46].

Finally, the impact of the “healthy worker effect” should be considered. Healthier individuals may be more likely to remain in demanding jobs, such as rotating-shift work, than unhealthy or less resilient workers [10,11]. This phenomenon could contribute to the more favorable lifestyle patterns observed among rotating-shift workers, particularly in physical activity, which was the only variable that remained significant after adjustment. Therefore, the apparent differences between groups may partly reflect underlying selection rather than a true effect of rotating-shift work. This is especially relevant given the cross-sectional design and the inclusion of a relatively healthy, normoglycemic working population. In addition, individual characteristics, such as relatively short cumulative exposure to night shifts, behavioral habits, and possible circadian adaptation, may also contribute to the observed findings.

### Strengths and limitations

Some limitations of this study should be acknowledged. First, the cross-sectional design prevented the inference of causal relationships. Second, our two groups differed in occupational profiles: the rotating-shift cohort primarily consisted of healthcare professionals, and the day-shift cohort had a broader mix of administrative and support staff, which may limit the generalizability of the findings. In addition, although information on exposure duration was available, the sample size limited the possibility of conducting more detailed analyses, such as dose–response assessments or sensitivity analyses.

A major strength of this study was the integration of clinical data (collected during the occupational health examinations) with data from validated questionnaires and structured interviews regarding physical activity, dietary habits, sleep quality, and chronotype. This approach provided a more integrated view of the workers’ health. Future research should employ longitudinal or prospective cohort designs to establish causal relationships and to identify factors that may enhance resilience in rotating-shift workers. Such studies should include more diverse occupational groups and consider potential mediating variables, such as duration of exposure to night work, sleep hygiene practices, and chronobiological adaptation.

## Conclusions

Rotating-shift work was not associated with an adverse cardiometabolic profile or an unhealthy lifestyle among normoglycemic hospital workers. Our findings suggest that individual and contextual factors may mitigate the potentially adverse effects of rotating-shift work on health. From a policy perspective, our findings suggest that, beyond limiting exposure to shift work, interventions should also focus on promoting protective behaviours such as physical activity and sleep hygiene. Furthermore, workplace environments may represent a key target for intervention, as contextual factors could contribute to enhancing workers’ resilience. In this context, actions such as improving access to healthy food, facilitating opportunities for physical activity, or adapting workplace lighting to better align with circadian rhythms could be considered. Longitudinal studies are needed to examine causal relationships and identify protective factors, such as physical activity and circadian adaptation, that may enhance the resilience of rotating-shift workers.

## Supporting information

S1 Table Bivariate analysis of the association of different variables with sex. *Abbreviations*: HDL: High-Density Lipoprotein; LDL: Low-Density Lipoprotein; BMI: Body Mass Index. An asterisk indicates a significant difference (p < 0.05) between groups. ^a^ Values are indicated as mean ± SD for continuous variables and n (%) for categorical variables. Independent t-test and χ^2^ tests were used to compare continuous and categorical variables, respectively. ^b^ The ‘Others’ category includes administrative assistant, administrative staff, social worker, psychologist, clinical psychology resident, pharmacist, pathology technician, biologist, telephone operator, management technician, and X-ray technician.
(DOCX)

S2 Table Adjusted general linear model results for clinical parameters. *Abbreviations*: HDL: High-Density Lipoprotein An asterisk indicates a significant difference (p < 0.05) between groups. ^a^ Values are presented as regression coefficients (β), 95% confidence intervals (CI), and p-values, and the model was adjusted for age, sex, Body Mass Index (BMI), and total physical activity.
(DOCX)

S3 Table General linear model analysis of the association of rotating-shift work with four clinical variables, stratified by age tertiles. *Abbreviations*: HDL: High-Density Lipoprotein. ^a^ Values are presented as regression coefficients (β), 95% confidence intervals (CI), and p-values, and each model was adjusted for age (tertiles), sex, Body Mass Index (BMI), and total physical activity.
(DOCX)

S4 Table General linear models for clinical variables including sex-by-shift work interaction. *Abbreviations*: HDL: High-Density Lipoprotein. An asterisk indicates a significant difference (p < 0.05) between groups. ^a^ Values are presented as regression coefficients (β), 95% confidence intervals (CI), and p-values. Models include main effects and a sex-by-shift work interaction term, adjusted for age.
(DOCX)

S5 Table Logistic regression analysis of cardiovascular risk (Framingham) adjusted for age and sex. *Abbreviations*: OR: odds ratio; CI: confidence interval; CVR: cardiovascular risk. An asterisk indicates a significant difference (p < 0.05) between groups. ^a^ Values are presented as regression coefficients (β), 95% confidence intervals (CI), and p-values, and the model was adjusted for age and sex.
(DOCX)

S6 Table General linear model analysis of the association of rotating-shift workers with total physical activity. An asterisk indicates a significant difference (p < 0.05) between groups. ^a^ Values are presented as regression coefficients (β), 95% confidence intervals (CI), and p-values, and the model was adjusted for age and sex.
(DOCX)

S7 Table Bivariate analysis of the association of different variables with circadian misalignment. *Abbreviations*: HDL: High-Density Lipoprotein; LDL: Low-Density Lipoprotein; BMI: Body Mass Index. An asterisk indicates a significant difference (p < 0.05) between groups. ^a^ Values are indicated as mean ± SD for continuous variables and n (%) for categorical variables. Independent t-test and χ^2^ tests were used to compare continuous and categorical variables, respectively. ^b^ Analysis restricted to participants with defined circadian alignment status (n = 98).
(DOCX)
