# Correction: Relationship of rotating-shift work with lifestyle and health status of hospital employees

**DOI:** 10.1371/journal.pgph.0007404

**Published:** 2026-09-29

**Authors:** Maria Antonia Alou-Soler, Aina M. Galmes-Panades, Ángel Arturo López-González, Aina M. Yañez, Miquel Bennasar-Veny

S1 Table was uploaded incorrectly. Please see the correct S1 Table below.

## Supporting information

S1 Table Bivariate analysis of the association of different variables with sex. *Abbreviations*: HDL: High-Density Lipoprotein; LDL: Low-Density Lipoprotein; BMI: Body Mass Index. An asterisk indicates a significant difference (p < 0.05) between groups. ^a^ Values are indicated as mean ± SD for continuous variables and n (%) for categorical variables. Independent t-test and χ^2^ tests were used to compare continuous and categorical variables, respectively. ^b^ The ‘Others’ category includes administrative assistant, administrative staff, social worker, psychologist, clinical psychology resident, pharmacist, pathology technician, biologist, telephone operator, management technician, and X-ray technician.
(DOCX)

## References

[1] Alou-Soler MA, Galmes-Panades AM, López-González ÁA, Yañez AM, Bennasar-Veny M. Relationship of rotating-shift work with lifestyle and health status of hospital employees. PLOS Glob Public Health. 2026;6(8):e0006956. doi: 10.1371/journal.pgph.0006956 42616748 PMC13489447

