# Supplementary material for: Relationship of rotating-shift work with lifestyle and health status of hospital employees
Source: PLOS Glob Public Health. 2026 Aug 19;6(8):e0006956. doi: 10.1371/journal.pgph.0006956 (PMC13489447; doi:10.1371/journal.pgph.0006956)
Supplement: Supplementary file 2 [file pgph.0006956.s002.docx]

**S2 Table** Adjusted general linear model results for clinical parameters

| **Variable** | **β** | **95% CI** | **p-value** |
| --- | --- | --- | --- |
| **BMI** |  |  |  |
| Fasting Plasma Glucose | 0.349 | 0.013, 0.684 | 0.042* |
| Systolic Blood Pressure | 0.816 | 0.444, 1.187 | <0.001* |
| Triglycerides | 2.138 | 1.034, 3.243 | <0.001* |
| HDL Cholesterol | -0.797 | -1.136, -0.458 | <0.001* |
| **Physical activity** |  |  |  |
| Fasting Plasma Glucose | 0.010 | -0.010, 0.030 | 0.311 |
| Systolic Blood Pressure | 0.009 | -0.014, 0.031 | 0.445 |
| Triglycerides | 0.013 | -0.053, 0.080 | 0.689 |
| HDL Cholesterol | 0.012 | -0.008, 0.032 | 0.253 |
| **Sex** |  |  |  |
| Fasting Plasma Glucose | 0.858 | -3.102, 4.817 | 0.670 |
| Systolic Blood Pressure | 9.016 | -13.40, -4.630 | <0.001* |
| Triglycerides | -25.84 | -38.87, -12.81 | <0.001* |
| HDL Cholesterol | 9.794 | 5.795, 13.793 | <0.001* |
| **Age** |  |  |  |
| Fasting Plasma Glucose | 0.199 | 0.041, 0.358 | 0.014* |
| Systolic Blood Pressure | 0.258 | 0.082, 0.434 | 0.004* |
| Triglycerides | 0.680 | 0.157, 1.202 | 0.011* |
| HDL Cholesterol | 0.062 | 0.099, 0.222 | 0.448 |

*Abbreviations*: HDL: High-Density Lipoprotein.

An asterisk indicates a significant difference (p < 0.05) between groups.

^a^ Values are presented as regression coefficients (β), 95% confidence intervals (CI), and p-values, and the model was adjusted for age, sex, Body Mass Index (BMI), and total physical activity.
