# Supplementary material for: Relationship of rotating-shift work with lifestyle and health status of hospital employees
Source: PLOS Glob Public Health. 2026 Aug 19;6(8):e0006956. doi: 10.1371/journal.pgph.0006956 (PMC13489447; doi:10.1371/journal.pgph.0006956)
Supplement: Supplementary file 3 [file pgph.0006956.s003.docx]

**S3 Table** General linear model analysis of the association of rotating-shift work with four clinical variables, stratified by age tertiles

| **Dependent variable** | **β** | **95% CI** | **p-value** |
| --- | --- | --- | --- |
| Fasting Plasma Glucose | −1.44 | −4.91, 2.03 | 0.415 |
| Systolic Blood Pressure | −2.44 | −6.29, 1.41 | 0.212 |
| Triglycerides | 0.07 | −11.30, 11.44 | 0.990 |
| HDL Cholesterol | 1.11 | −2.38, 4.61 | 0.531 |

*Abbreviations*: HDL: High-Density Lipoprotein

^a^ Values are presented as regression coefficients (β), 95% confidence intervals (CI), and p-values, and each model was adjusted for age (tertiles), sex, Body Mass Index (BMI), and total physical activity.
