# Supplementary material for: Relationship of rotating-shift work with lifestyle and health status of hospital employees
Source: PLOS Glob Public Health. 2026 Aug 19;6(8):e0006956. doi: 10.1371/journal.pgph.0006956 (PMC13489447; doi:10.1371/journal.pgph.0006956)
Supplement: Supplementary file 4 [file pgph.0006956.s004.docx]

**S4 Table** General linear models for clinical variables, including sex-by-shift work interaction

| **Dependent variable** | **β** | **95% IC** | **p-value** |
| --- | --- | --- | --- |
| ***Fasting Plasma Glucose*** |  |  |  |
| Shift Work | 0.11 | -3.69, 3.90 | 0.179 |
| Sex | 3.78 | -2.60, 10.15 | 0.244 |
| Shift Work × Sex | -5.44 | -13.39, 2.52 | 0.179 |
| Age | 0.24 | 0.09, 0.39 | 0.003* |
| ***Systolic Blood Pressure*** |  |  |  |
| Shift Work | -1.38 | -5.72, 297 | 0.533 |
| Sex | 13.66 | 6.36, 20.95 | <0.001* |
| Shift Work × Sex | -3.81 | -12.91, 5.30 | 0.411 |
| Age | 0.36 | 0.18, 0.53 | <0.001* |
| ***Triglycerides*** | | | |
| Shift Work \| 0.18 \| -12.62, 12.99 \| 0.977; Sex \| 32.43 \| 10.91, 53.94 \| 0.003*; Shift Work × Sex \| -1.74 \| -28.59, 25.12 \| 0.899; Age \| 0.94 \| 0.42, 1.46 \| <0.001*; ***HDL-cholesterol*** \| \| \|; Shift Work \| 1.82 \| -2.20, 5.84 \| 0.373; Sex \| -12.22 \| -18.97, -5.47 \| <0.001*; Shift Work × Sex \| 1.42 \| -7.01, 9.85 \| 0.740; Age \| -0.04 \| -0.20, 0.13 \| 0.674 | | | |

*Abbreviations*: HDL: High-Density Lipoprotein

An asterisk indicates a significant difference (p < 0.05) between groups.

^a^ Values are presented as regression coefficients (β), 95% confidence intervals (CI), and p-values. Models include main effects and a sex-by-shift work interaction term, adjusted for age.
