# Supplementary material for: Relationship of rotating-shift work with lifestyle and health status of hospital employees
Source: PLOS Glob Public Health. 2026 Aug 19;6(8):e0006956. doi: 10.1371/journal.pgph.0006956 (PMC13489447; doi:10.1371/journal.pgph.0006956)
Supplement: Supplementary file 5 [file pgph.0006956.s005.docx]

**S5 Table** Logistic regression analysis of cardiovascular risk (Framingham) adjusted for age and sex.

| **Variable** | **OR** | **95% CI** | **p-value** |
| --- | --- | --- | --- |
| Rotating-shift work  Yes  No | 1.18  - | 0.20, 6.82 | 0.857 |
| Age | 1.65 | 1.34, 2.04 | <0.001* |

*Abbreviations*: OR: odds ratio; CI: confidence interval; CVR: cardiovascular risk.

An asterisk indicates a significant difference (p < 0.05) between groups.

^a^ Values are presented as Odds Ratios (OR), 95% confidence intervals (CI), and p-values, and the model was adjusted for age and sex.
