# Supplementary material for: Relationship of rotating-shift work with lifestyle and health status of hospital employees
Source: PLOS Glob Public Health. 2026 Aug 19;6(8):e0006956. doi: 10.1371/journal.pgph.0006956 (PMC13489447; doi:10.1371/journal.pgph.0006956)
Supplement: Supplementary file 6 [file pgph.0006956.s006.docx]

**S6 Table** General linear model analysis of the association of rotating-shift workers with total physical activity

| **Variable** | **β** | **95% CI** | **p-value** |
| --- | --- | --- | --- |
| Rotating-shift work  Yes  No | 29.76  - | 6.68, 52.84 | 0.012* |
| Sex  Male  Female | 31.35  - | 5.91, 56.79 | 0.016* |
| Age | -0.06 | -1.08, 0.97 | 0.916 |

An asterisk indicates a significant difference (p < 0.05) between groups.

^a^ Values are presented as regression coefficients (β), 95% confidence intervals (CI), and p-values, and the model was adjusted for age and sex.
