# Supplementary material for: Relationship of rotating-shift work with lifestyle and health status of hospital employees
Source: PLOS Glob Public Health. 2026 Aug 19;6(8):e0006956. doi: 10.1371/journal.pgph.0006956 (PMC13489447; doi:10.1371/journal.pgph.0006956)
Supplement: Supplementary file 7 [file pgph.0006956.s007.docx]

**S7 Table** Bivariate analysis of the association of different variables with circadian misalignment

| **Variables** | **All sample n=98** | **Alignment n=52** | **Misalignment n=46** | **p-value** |
| --- | --- | --- | --- | --- |
|  | Mean ± SD / N(%) |  |  |  |
| ***Sociodemographic and occupational factors*** | | | | |
| Age (years) | 42.29±11.19 | 44.39±10.90 | 42.50±10.05 | 0.307 |
| Sex  Woman  Men  Night Shift Work  Yes  No | 176 (78.2)  49 (21.8)  123 (54.7)  102 (45.3) | 34 (73.9)  12 (26.1)  11 (23.9)  35 (76.1) | 40 (76.9)  12 (23.1)  40 (76.9)  12 (23.1) | 0.644  <0.001* |
| ***Clinical parameters*** |  |  |  |  |
| Fasting Plasma Glucose (mg/dL) | 88.83±12.17 | 89.04±9.17 | 90.40±17.96 | 0.517 |
| Total Cholesterol (mg/dL) | 187.82±35.19 | 190.76±32.75 | 181.25±35.45 | 0.302 |
| HDL Cholesterol (mg/dL) | 59.41±13.31 | 58.57±14.33 | 59.37±12.95 | 0.877 |
| LDL Cholesterol (mg/dL) | 113.48±32.14 | 116.59±30.13 | 107.60±31.17 | 0.307 |
| Triglycerides (mg/dL) | 77.67±42.73 | 78.02±39.78 | 76.38±37.46 | 0.970 |
| ***Anthropometric variables*** |  |  |  |  |
| BMI (kg/m²)  Waist Circumference (cm) | 24.49±4.99  79.78±12.60 | 24.88±5.30  81.55±12.64 | 25.51±5.19  80.60±14.38 | 0.133  0.390 |
| ***Lifestyle factors*** | | | | |
| Adherence to Mediterranean Diet | 8.68±1.90 | 8.57±1.92 | 8.88±2.11 | 0.666 |
| Light Physical Activity (min/day) | 1.43±6.02 | 1.12±4.75 | 1.40±8.93 | 0.912 |
| Moderate Physical Activity (min/day) | 44.07±63.59 | 44.21±74.74 | 49.29±62.77 | 0.780 |
| Vigorous Physical Activity (min/day) | 20.25±40.86 | 10.75±18.71 | 30.95±59.58 | 0.046* |
| Total Physical Activity (min/day) | 65.76±81.33 | 56.07±80.40 | 81.63±93.69 | 0.247 |
| Smoking  Non-smoker  Smoker  Former Smoker | 139 (61.8)  35 (15.6)  51 (22.7) | 33 (71.7)  3 (6.5)  10 (21.7) | 32 (61.5)  5 (9.6)  15 (28.8) | 0.078 |
| ***Cardiovascular Risk (Framingham)*** | | | | |
| Cardiovascular Risk  Low  Intermediate - High | 205 (91.1)  20 (8.9) | 45 (97.8)  1 (2.2) | 47 (90.4)  5 (9.6) | 0.191 |
| ***Sleep factors*** | | | | |
| Sleep Quality  Good  Bad | 101 (44.9)  124 (55.1) | 21 (45.7)  25 (54.3) | 24 (46.2)  28 (53.8) | 0.962 |

*Abbreviations*: HDL: High-Density Lipoprotein; LDL: Low-Density Lipoprotein; BMI: Body Mass Index.

An asterisk indicates a significant difference (p < 0.05) between groups.

^a^ Values are indicated as mean ± SD for continuous variables and n (%) for categorical variables. Independent t-test and χ^2^ tests were used to compare continuous and categorical variables, respectively.

^b^ Analysis restricted to participants with defined circadian alignment status (n=98).
