# Supplementary material for: Relationship of rotating-shift work with lifestyle and health status of hospital employees
Source: PLOS Glob Public Health. 2026 Aug 19;6(8):e0006956. doi: 10.1371/journal.pgph.0006956 (PMC13489447; doi:10.1371/journal.pgph.0006956)
Supplement: Supplementary file 8 [file pgph.0006956.s009.docx]

Dear Editor,

We sincerely thank you and the reviewers for the time and effort dedicated to the evaluation of our manuscript entitled *“Relationship of rotating-shift work with lifestyle and health status of hospital employees.”*

We have carefully addressed all the reviewers’ comments and revised the manuscript accordingly. We greatly appreciate the constructive feedback provided, which has helped us to improve the quality and clarity of our work.

We are grateful for the opportunity to revise our manuscript and for your consideration of our work for publication in *PLOS Global Public Health*.

Yours sincerely,

Aina M Galmés Panadés, PhD

On behalf of all authors

**Response to reviewers**

**Reviewer #1:** This is a well-structured and relevant study addressing an important occupational health question. The integration of validated behavioral instruments with objective clinical data is a strength.

**R**: We thank Reviewer #1 for the useful suggestions.

Minor revisions are suggested:

1. Briefly justify covariate selection in adjusted models or acknowledge potential residual confounding (e.g., occupational differences).

**R**: Thank you for pointing this out. Covariates (age, sex, body mass index, and total physical activity) were selected based on prior evidence and their potential role as confounders in the relationship between shift work and cardiometabolic outcomes. We have now clarified this in the Methods section. In addition, we acknowledge that residual confounding due to unmeasured or insufficiently adjusted factors—such as occupational differences between groups—may still be present, and this has been explicitly noted in the manuscript (page 8-9, lines 191-193).

2. Clearly define circadian misalignment in the Methods section.

**R**: Thank you for this comment. We have clarified the definition of circadian alignment/misalignment in the Methods section. In this study, circadian alignment/misalignment was defined based on the concordance between individual chronotype and work schedule. Chronotype was assessed using the Morningness–Eveningness Questionnaire (MEQ). Alignment was defined as a match between chronotype and work schedule (i.e., morning chronotype in day-shift workers and evening chronotype in rotating-shift workers), whereas misalignment was defined as a mismatch (i.e., evening chronotype in day-shift workers and morning chronotype in rotating-shift workers). Additionally, participants with intermediate chronotype were excluded from this analysis to ensure a clear classification. These clarifications have been incorporated into the Methods section (pages 8, lines 171-180).

3. Slightly soften wording suggesting better health among rotating-shift workers, given the cross-sectional design.

**R**: We appreciate this comment. We have revised the wording throughout the manuscript to ensure a more cautious and neutral interpretation of the findings, avoiding any implication of a protective or causal effect of rotating-shift work. In particular, statements suggesting better health among rotating-shift workers have been rephrased to emphasize the absence of significant associations after adjustment and the cross-sectional nature of the study. These changes have been applied in the Abstract, Discussion, and Conclusions sections.

4. Confirm statistical assumption checks.

**R**: We have added a sentence in the Statistical Analyses section confirming that the assumptions underlying the statistical tests were verified (page 8, line 186-187). For continuous variables, normality was assessed using the Shapiro-Wilk test and homogeneity of variances with Levene's test prior to applying independent samples t-tests. For the GLM, residual normality and homoscedasticity were also checked before reporting results.

5. Ensure compliance with PLOS data availability requirements.

**R**: We thank the reviewer for this important comment. In accordance with PLOS data sharing policies, the dataset has been deposited in the Dryad Digital Repository.

The dataset has been assigned a permanent DOI (doi:10.5061/dryad.fn2z34v9j) and is currently available in "Private for Peer Review" mode. A secure reviewer access link is provided below to allow access during the peer-review process: <http://datadryad.org/share/LINK_NOT_FOR_PUBLICATION/NOt9TSxvbNp5T2G0dYE_XG0DV_OpMpndE8QZGyUxalU>

The Data Availability Statement has been updated accordingly.

6. Correct minor typographical errors.

**R**: Minor typographical and formatting errors have been carefully reviewed and corrected throughout the manuscript to improve clarity and consistency.

**Reviewer #2:** The manuscript has many strengths. It addresses an important occupational, cardiovascular and public health concern, used validated instruments and standardized clinical measurements, and reported following sound ethical principles. The inclusion of multiple lifestyle and cardiometabolic variables in a a good sample of healthcare worker population is a notable strength and provides useful descriptive insights.

**R**: We thank Reviewer #2 for this valuable suggestion.

However, a crucial limitation that impacts the most favorable review is that the conclusions seem to overstate the findings based on the current data presented. Of most importance is that after adjustment for confounders, most cardiometabolic outcomes were not significantly associated with rotating-shift work, yet broader claims about better health status were made for shift workers. The cross-sectional design limits causal inference, and the restriction of the study participants to only normoglycemic workers, and in one hospital may reduce generalizability.

The manuscript briefly mentions the healthy worker effect, but this issue likely plays an important role in the observed differences between groups. The shift workers shifts may represent a healthier subset of employees, while unhealthier individuals may transition to the day shift. This could partially explain the more favorable lifestyle, particularly in physical activity, the only variable that remained statistically significant. This possibility should be discussed more explicitly.

The manuscript would benefit from substantially revising the conclusions to align with the adjusted results. This will improve clarity and transparency of the statistical analyses, and more fully address confounding and study limitations.

**R**: We thank the reviewer for highlighting this important issue. We have revised the manuscript to ensure a more cautious interpretation of the findings, particularly by aligning the conclusions with the adjusted analyses and avoiding any implication of a protective effect of rotating-shift work. The cross-sectional design and its limitations for causal inference are now more explicitly acknowledged.

In addition, we have substantially expanded the discussion of the healthy worker effect. We now explicitly acknowledge that rotating-shift workers in our sample were younger and had a different job distribution compared to day-shift workers, and that healthier individuals may self-select into or remain in rotating-shift positions, while those with worse health may transfer to day work. This selection mechanism could plausibly explain the more favorable physical activity profile and some of the unadjusted cardiometabolic differences observed (please see page 21 of the manuscript).

These aspects, together with the limited generalizability due to the inclusion of normoglycemic workers from a single institution, are now more prominently discussed in the revised manuscript (Discussion and Conclusions sections).

**Reviewer #3:** This publication examines a significant and relevant issue in occupational health: the correlation between rotating-shift work and cardiometabolic and lifestyle outcomes among hospital personnel. The amalgamation of clinical indicators with established behavioural and sleep-related instruments is praiseworthy and enhances the study's usefulness.
However, the manuscript must address some significant methodological and interpretation concerns before it can be considered for publication.

**R**: We thank reviewer #3 for the valuable comments.

Principal Observations

1. Research methodology and causal inference

The cross-sectional design fundamentally restricts causal inference. The text often suggests that rotating-shift work has protective benefits (e.g., healthier lifestyle, reduced cardiovascular risk), a claim not supported by the study design. These interpretations should be amended to indicate association solely, and the potential for reverse causality must be expressly addressed.

**R**: We appreciate this comment. We have revised the wording throughout the manuscript to ensure a more cautious and neutral interpretation of the findings, avoiding any implication of causality and consistently referring to associations only.

In addition, we have explicitly acknowledged the limitations inherent to the cross-sectional design, including the potential for reverse causality, whereby healthier individuals may be more likely to remain in or be assigned to rotating-shift positions. These considerations have been incorporated into the Abstract, Discussion, and Conclusions sections.

2. Healthy worker effect and selection bias

A significant issue is the probability of a pronounced healthy worker effect. The rotating-shift cohort is markedly younger and has a distinct vocational makeup compared with day workers. These discrepancies are likely to skew the results in favour of a more advantageous health profile among shift workers.

The existing adjustment technique (age, sex, BMI, physical activity) is inadequate to completely mitigate this bias. The authors ought to contemplate:

Stratified analyses (e.g., by age or professional category), or

More sophisticated adjustment methodologies (e.g., propensity score techniques)

This limitation must be significantly broadened in the discourse.

**R**: We thank the reviewer for this insightful comment regarding the potential influence of a healthy worker effect and selection bias. In response, we conducted additional analyses stratifying the sample by age tertiles to explore whether the observed associations were consistent across age groups. These analyses showed no significant association between rotating-shift work and cardiometabolic outcomes across age strata (see Supplementary 3 Table).

We acknowledge, however, that this approach does not fully eliminate the possibility of residual selection bias. More advanced methods, such as propensity score techniques, were not applied due to the limited sample size, which may compromise model stability.

In addition, we have substantially expanded the discussion of the healthy worker effect, explicitly addressing differences in age and occupational distribution between groups and their potential influence on the observed results.

3. Remaining confounding factors

Significant confounders were insufficiently addressed, including:

Duration and cumulative exposure to nocturnal work shifts

Socioeconomic status

Workload and occupational stress

Comprehensive sleep scheduling and circadian disturbance

These characteristics are very pertinent to shift work studies and may considerably affect the results.

**R**: We thank the reviewer for this important comment. In response, we performed additional analyses incorporating duration of rotation-shift work (years of exposure) as a proxy for cumulative exposure, and educational level (university vs. non-university) as a proxy for socioeconomic status. These variables were included in the multivariable models as potential confounders, and the results remained consistent with the findings.

We acknowledge that other relevant factors, such as workload, occupational stress, and detailed sleep scheduling, were not directly measured and may have contributed to residual confounding. This limitation has been explicitly acknowledged in the Discussion.

In addition, we included supplementary analyses exploring circadian alignment/misalignment (see Supplementary 7 Table), as a proxy of circadian disturbance, to provide further context for the interpretation of the findings.

Definition of exposure

The definition of "rotating-shift work" is inadequately detailed. The characterisation is ambiguous:

Frequency and intensity of nocturnal shifts

Length of exposure (years)

Rotational patterns

The absence of this information constrains the interpretation of the findings. If data are accessible, dose–response analyses ought to be contemplated.

**R**: We thank the reviewer for this important comment. We have clarified and expanded the definition of rotating-shift work in the Methods section to provide a more detailed characterization.

Specifically, we now describe the forward rotation schedule (morning, afternoon, and night shifts), the average frequency of night shifts (approximately 4–6 per month), and the collection of data on years of exposure to rotating-shift work.

We acknowledge that, although information on exposure duration was available, dose–response analyses were not conducted due to the limited sample size, which restricted the ability to perform more detailed stratified analyses. This limitation has been acknowledged in the Discussion.

5. Analysis of Results

The observation that rotating-shift workers display superior cardiometabolic profiles contradicts extensive epidemiological research. This gap is probably attributable to prejudice (e.g., selection bias, residual confounding) rather than an actual protective effect.

The discourse requires revision to:

Refrain from excessive interpretation

Conduct a more rigorous comparison with current meta-analyses.

Highlight alternate interpretations (e.g., behavioural compensation, selection biases)

**R**: We thank the reviewer for this insightful comment. We agree that our findings should be interpreted with caution, particularly in light of the existing epidemiological evidence. We have revised the Discussion to avoid overinterpretation and to ensure that our results are presented as associations rather than indicative of any protective effect.

We have also strengthened the comparison with previous studies and recent meta-analyses, highlighting both consistencies and discrepancies with the existing literature. In addition, we now more explicitly discuss alternative explanations for our findings, including selection bias (healthy worker effect), residual confounding, and potential behavioral compensation (e.g., higher physical activity and structured lifestyle patterns among rotating-shift workers).

These aspects have been addressed in the Discussion section (lines 408-421 and 349-393) to provide a more balanced and contextualized interpretation of the results.

6. Statistical Examination

The application of GLM is suitable; nonetheless, the statistical analysis is deficient in detail and rigour.

The model assumptions are not disclosed.

No interaction analyses, such as sex-by-shift work, are provided.

No sensitivity analyses were performed.

These issues should be resolved to enhance the validity of the findings.

**R**: We thank the reviewer for this important comment. Additional details regarding the statistical approach have been included in the Methods section. Parametric tests were used only when the normality assumption was satisfied, and assumptions for the GLM, including residual normality and homoscedasticity, were also verified prior to analysis.

We conducted the proposed interaction analyses to assess whether the association between rotating-shift work and cardiometabolic outcomes differed by sex. No statistically significant interactions were observed, and the results are presented in Supplementary 4 Table.

Sensitivity analyses were not performed due to the limited sample size, which restricted the feasibility of additional stratified or robustness analyses without compromising statistical validity. This limitation has been acknowledged in the manuscript.

7. Sample Size and Statistical Power

The limited sample size (n = 225) may constrain statistical power, especially for subgroup analysis. A rationale for the sample size or a declaration of reduced statistical power is advisable.

**R**: We thank the reviewer for this valuable comment. In response, we have added a new subsection in the Methods (“Sample size and statistical power”, line 199-207) to provide a rationale for the sample size, including the minimum detectable effect size under standard assumptions (α = 0.05, 80% power). We also acknowledge that the relatively limited sample size may have reduced the statistical power to detect small effects, particularly in subgroup analyses. This limitation has been explicitly stated in the manuscript.

8. Accessibility of Data

The manuscript indicates that data are accessible "upon request", which contravenes PLOS data sharing policy. The authors must either upload the dataset to a public repository or provide a clear justification for any limits imposed.

**R**: We thank the reviewer for highlighting this issue. In accordance with PLOS data sharing policies, the dataset has been deposited in the Dryad Digital Repository.

The dataset has been assigned a permanent DOI (doi:10.5061/dryad.fn2z34v9j) and is currently available in "Private for Peer Review" mode. A secure reviewer access link is provided below to allow access during the peer-review process: http://datadryad.org/share/LINK_NOT_FOR_PUBLICATION/NOt9TSxvbNp5T2G0dYE_XG0DV_OpMpndE8QZGyUxalU

The Data Availability Statement has been updated accordingly.

Marginal Observations

Minor typographical problems exist (e.g., repetition of “rotating rotating-shift”).

Tables would be enhanced by incorporating effect sizes alongside p-values.

The abstract should avoid causal language and more accurately reflect the cross-sectional nature of the study.

Recent literature from 2023 to 2025 should be integrated to enhance the discourse.

**R**: We thank the reviewer for these valuable suggestions. Effect size measures have been incorporated into the tables alongside p-values, with regression coefficients (β) and odds ratios (OR) reported together with their corresponding 95% confidence intervals. Minor typographical errors have been carefully revised and corrected.

The abstract has also been reviewed and adapted according to the reviewer’s recommendations, avoiding causal language and more accurately.

Regarding the inclusion of more recent literature (2023–2025), we note that the majority of the references included in the Discussion are already recent (2020–2025), and we did not identify additional studies that would substantially modify the interpretation of our findings.

Final Assessment

This manuscript possesses potential and tackles a pertinent public health concern. Significant modifications are necessary, especially regarding methodological rigour, bias control, and the interpretation of findings. I suggest a substantial change prior to additional evaluation.

**R**: We thank the reviewer for the thorough evaluation and constructive feedback. We have carefully addressed all the comments and implemented substantial revisions throughout the manuscript to improve the methodological rigour, better account for potential sources of bias, and ensure a more cautious and accurate interpretation of the findings.

**Reviewer #4:** Generally, the manuscript is well articulated and meets the standards for publication.
It provides insights on an emerging issue that needs to be addressed by policy makers in the health sector.

**R**: We thank Reviewer #4 for the useful comments.

The Authors could however consider the following comments:
1. Line 88, delete the word rotating and remain with rotating-shift.
2. Line 99, instead of using the term discrepant, use the word different/contrary.
3. Line 113, I suggest including the name of the institution and not using the term single institution in Spain.

**R**: We thank the reviewer for these helpful comments. The manuscript has been revised accordingly, including correction of wording and explicit specification of the study institution in the Methods section.

4. In the study design, explain how the study size was arrived at. What sampling technique was used and the assumptions.

**R**: We thank the reviewer for this valuable comment. The Methods section has been revised to clarify the sampling approach and the basis for the study size. Participants were recruited using a convenience sampling strategy from hospital employees undergoing routine occupational health assessments.

We have also clarified that the sample size was determined by the number of eligible participants available during the study period, rather than by a priori sample size calculation. In addition, a subsection on sample size and statistical power has been included (“Sample size and statistical power”) to provide a post hoc assessment of the study’s ability to detect clinically meaningful differences under standard assumptions.

5. Line 151, amend to were also assessed during this examination.

**R**: Thank you, done.

6. In the tables 1.1 and 1.2, I suggest to add the N(%) across the columns.

**R**: We appreciate the reviewer for the helpful suggestions. The manuscript has been revised accordingly.

7. I have concerns on the p-values reported and the tests used in Tables 1.1 and 1.2:
A one-way ANOVA is used to determine if there are statistically significant differences between the means of three or more independent (unrelated) groups. It evaluates the impact of a single factor—a categorical independent variable—on a continuous dependent variable.
I recommend to use independent t-test to compare the groups.
Also, for the p-values reported for Job Category, it would be inaccurate to report the chi-square for that since some of the cell frequencies are less than 5. I suggest to use the Fishers exact test.

And also for Cardiovascular Risk, some of the cells are less than 5. Therefore, chi-square will not be accurate for the p-value estimate.

**R**: We thank the reviewer for these valuable comments. Following the suggestion, one-way ANOVA was replaced by independent samples t-test for comparisons between rotating-shift and day-shift workers, as only two groups were analyzed. The results remained unchanged.

For categorical variables, we reassessed the assumptions of the chi-square test by examining expected cell counts. For most variables (e.g., job category), all expected frequencies met the required assumptions, supporting that the use of Pearson’s chi-square test was appropriate.

However, for the Cardiovascular Risk variable (Framingham), some expected cell counts in the original three-category classification were below 5, which did not comply with the chi-square assumptions. Therefore, this variable was recoded into a binary category (low vs. moderate-high risk), and comparisons were performed using Fisher’s exact test. The results remained statistically significant (p = 0.002) and consistent with the original findings. These changes have been incorporated into the revised manuscript, including updates in the Methods section and table footnotes.

8. Line 196, can we really talk of more likely for an association test? Likelihoods are estimated using a regression analysis. I suggest you say "rotating-shift workers were majority women".

**R**: Thank you, done.

9. Line 328, I suggest use the word differences.

**R**: We thank the reviewer for the helpful comments. The manuscript has been revised to address the wording issues, and the institution has been explicitly specified as recommended.

10. On the conclusion, what can policy makers deduce from this study? A statement from the researchers can come along away.

You could think around:

i. Shift to Health Promotion over Risk Avoidance: Instead of just limiting hours, policy should incentivize programs that encourage physical activity and sleep hygiene, as these specific factors appear to "enhance the resilience" of the workforce.

ii. Context is a Lever for Intervention: Since individual and contextual factors mitigate risk, policy should focus on workplace environments (e.g., providing healthy food options, gym access, or optimized lighting for circadian adaptation) to build worker resilience.

**R**: We thank the reviewer for the useful suggestions. The proposed policy-oriented implications have been incorporated into the Conclusions section of the revised manuscript.

From a policy perspective, our findings suggest that, beyond limiting exposure to shift work, interventions may benefit from focusing on promoting protective behaviors such as physical activity and sleep hygiene. Furthermore, workplace environments may represent a key target for intervention, as contextual factors could contribute to enhancing workers’ resilience. In this context, actions such as improving access to healthy food, facilitating opportunities for physical activity, or adapting workplace lighting to better align with circadian rhythms could be considered.
