# Supplementary material for: Relationship of rotating-shift work with lifestyle and health status of hospital employees
Source: PLOS Glob Public Health. 2026 Aug 19;6(8):e0006956. doi: 10.1371/journal.pgph.0006956 (PMC13489447; doi:10.1371/journal.pgph.0006956)
Supplement: Supplementary file 9 [file pgph.0006956.s010.docx]

**Review Comments to the Author**

Please use the space provided to explain your answers to the questions above. You may also include additional comments for the author, including concerns about dual publication, research ethics, or publication ethics. (Please upload your review as an attachment if it exceeds 20,000 characters)

Reviewer #2: Dear Authors,

You have done an excellent job with addressing the reviewer concerns/comments. Your point by point response to all comments is commendable. Keep up the good work.

R: We thank the reviewer for their positive assessment of our manuscript and for their encouraging comments.

Reviewer #4: Thanks to the authors for their extensive changes in the manuscript. There has been a significant improvement in the new version in terms of methodological clarity, statistical strength, interpretation of findings and overall presentation.
In particular, the authors have:

1. Clarified the exposure definition and rotating-shift schedule,
2. Revised the statistical analyses appropriately (including replacement of ANOVA with independent t-tests where applicable and use of Fisher’s exact test when assumptions were violated)
3. Clarified model assumptions and interaction analyses,
4. strengthened the interpretation of findings and policy implications, and complied with PLOS data availability requirements through deposition in Dryad.
5. The revised conclusions are now substantially more cautious and appropriately framed for a cross-sectional study.

I only have a minor remaining concern regarding the methodology. The authors have noted that they did not pre-calculate the sample size, but they should make this clear and be consistent across the manuscript in the Method section, indicating that the analyses they present were based on a post hoc power/detectable effect estimate and not a pre-calculated sample size.
Additionally, they state that the sample was 240, but what is reported in the tables is 225. I suggest you include the response rate in the results section. As it stands, it raises questions about whether the 15 excluded participants introduce selection bias and whether the study remains adequately powered at n=225 rather than n=240.

Overall, the manuscript, as a whole, has been greatly improved and is now scientifically acceptable. If the description of the sample size is clarified, it should suffice.

**R**: We thank the reviewer for this helpful comment. We have revised the Sample Size section to explicitly clarify that no a priori sample size calculation was performed, and a post hoc assessment of statistical power and detectable effect size was performed. We clarified in the Sample Size section, “*No sample size calculation was performed before participant recruitment. As a post hoc assessment, we estimated that a sample of 225 participants would provide adequate statistical power to detect clinically meaningful between-group differences in Framingham cardiovascular risk*”.

Fifteen participants were excluded due to incomplete data required for the analyses; to avoid inconsistencies, we have clarified in the Sample Size section that the final analytical sample consisted of 225 participants. The Results section has also been revised to explicitly state that a total of 240 individuals participated in the study, of whom 225 had valid data available for the analyses. To address the reviewer’s concern, we consistently report the final analytical sample size (n = 225) throughout the manuscript.

Regarding statistical power, the detectable effect size was recalculated using the final analytical sample (n = 225). The Sample Size section was updated accordingly: “… *we estimated that a sample of 225 participants would provide adequate statistical power to detect clinically meaningful between-group differences in Framingham cardiovascular risk. Assuming a two-sided alpha level of 0.05, 80% power, and approximately equal group sizes, a sample of 225 participants would allow the detection of a standardized mean difference of approximately 0.37, corresponding to a small-to-moderate effect size. Considering a plausible standard deviation of 7–8 percentage points for the Framingham risk score, this translates into the ability to detect absolute differences of approximately 2.6–3.0 percentage points between groups*”.
