# Supplementary material for: Relationship of rotating-shift work with lifestyle and health status of hospital employees
Source: PLOS Glob Public Health. 2026 Aug 19;6(8):e0006956. doi: 10.1371/journal.pgph.0006956 (PMC13489447; doi:10.1371/journal.pgph.0006956)
Supplement: Peer review history

# Peer review: Decision Letter 0

**DOI:** 10.1371/journal.pgph.0006956.r001

**Authors:** Feten Fekih-Romdhane

23 Mar 2026

PGPH-D-25-03895

Relationship of rotating-shift work with lifestyle and health status of hospital employees

PLOS Global Public Health

Dear Dr. Galmes-Panades,

Thank you for submitting your manuscript to PLOS Global Public Health. After careful consideration, we feel that it has merit but does not fully meet PLOS Global Public Health’s publication criteria as it currently stands. Therefore, we invite you to submit a revised version of the manuscript that addresses the points raised during the review process.

Please submit your revised manuscript by May 07 2026 11:59PM. If you will need more time than this to complete your revisions, please reply to this message or contact the journal office at globalpubhealth@plos.org. When you're ready to submit your revision, log on to https://www.editorialmanager.com/pgph/ and select the 'Submissions Needing Revision' folder to locate your manuscript file.

Please include the following items when submitting your revised manuscript:

- A letter that responds to each point raised by the editor and reviewer(s). You should upload this letter as a separate file labeled 'Response to Reviewers'.
- A marked-up copy of your manuscript that highlights changes made to the original version. You should upload this as a separate file labeled 'Revised Manuscript with Track Changes'.
- An unmarked version of your revised paper without tracked changes. You should upload this as a separate file labeled 'Manuscript'.

Guidelines for resubmitting your figure files are available below the reviewer comments at the end of this letter.

We look forward to receiving your revised manuscript.

Kind regards,

Feten Fekih-Romdhane

Academic Editor

PLOS Global Public Health

**Journal Requirements:**

1. Please amend your detailed Financial Disclosure statement. This is published with the article. It must therefore be completed in full sentences and contain the exact wording you wish to be published.

i. Please clarify all sources of financial support for your study. List the grants, grant numbers, and organizations that funded your study, including funding received from your institution. Please note that suppliers of material support, including research materials, should be recognized in the Acknowledgements section rather than in the Financial Disclosure.

ii. State the initials, alongside each funding source, of each author to receive each grant. For example: "This work was supported by the National Institutes of Health (####### to AM; ###### to CJ) and the National Science Foundation (###### to AM)."

iii. State what role the funders took in the study. If the funders had no role in your study, please state: “The funders had no role in study design, data collection and analysis, decision to publish, or preparation of the manuscript.”

iv. If any authors received a salary from any of your funders, please state which authors and which funders.

2. In the online submission form, you indicated that “The data set will be avaiable upon request”.

All PLOS journals now require all data underlying the findings described in their manuscript to be freely available to other researchers, either

1. In a public repository,

2. Within the manuscript itself, or

3. Uploaded as supplementary information.

This policy applies to all data except where public deposition would breach compliance with the protocol approved by your research ethics board. If your data cannot be made publicly available for ethical or legal reasons (e.g., public availability would compromise patient privacy), please explain your reasons by return email and your exemption request will be escalated to the editor for approval. Your exemption request will be handled independently and will not hold up the peer review process, but will need to be resolved should your manuscript be accepted for publication. One of the Editorial team will then be in touch if there are any issues.

3. Please ensure that your Ethics Statement is available in its entirety at the beginning of your Methods section, under a subheading 'Ethics Statement'.

4. We noticed that you used “data not shown” in the manuscript. We do not allow these references, as the PLOS data access policy requires that all data be either published with the manuscript or made available in a publicly accessible database. Please amend the supplementary material to include the referenced data or remove the references.

If the reviewer comments include a recommendation to cite specific previously published works, please review and evaluate these publications to determine whether they are relevant and should be cited. There is no requirement to cite these works unless the editor has indicated otherwise.

**Additional Editor Comments (if provided):**

[Note: HTML markup is below. Please do not edit.]

Reviewers' comments:

Reviewer's Responses to Questions

**Comments to the Author**

1. Does this manuscript meet PLOS Global Public Health’s publication criteria? Is the manuscript technically sound, and do the data support the conclusions? The manuscript must describe methodologically and ethically rigorous research with conclusions that are appropriately drawn based on the data presented.

Reviewer #1: Yes

Reviewer #2: Partly

Reviewer #3: Partly

Reviewer #4: Yes

**********

2. Has the statistical analysis been performed appropriately and rigorously?

Reviewer #1: I don't know

Reviewer #2: Yes

Reviewer #3: No

Reviewer #4: No

**********

3. Have the authors made all data underlying the findings in their manuscript fully available (please refer to the Data Availability Statement at the start of the manuscript PDF file)?

The PLOS Data policy requires authors to make all data underlying the findings described in their manuscript fully available without restriction, with rare exception. The data should be provided as part of the manuscript or its supporting information, or deposited to a public repository. For example, in addition to summary statistics, the data points behind means, medians and variance measures should be available. If there are restrictions on publicly sharing data—e.g. participant privacy or use of data from a third party—those must be specified.

Reviewer #1: No

Reviewer #2: No

Reviewer #3: No

Reviewer #4: No

**********

4. Is the manuscript presented in an intelligible fashion and written in standard English?

PLOS Global Public Health does not copyedit accepted manuscripts, so the language in submitted articles must be clear, correct, and unambiguous. Any typographical or grammatical errors should be corrected at revision, so please note any specific errors here.

Reviewer #1: Yes

Reviewer #2: Yes

Reviewer #3: Yes

Reviewer #4: Yes

**********

5. Review Comments to the Author

Please use the space provided to explain your answers to the questions above. You may also include additional comments for the author, including concerns about dual publication, research ethics, or publication ethics. (Please upload your review as an attachment if it exceeds 20,000 characters)

**Reviewer #1:**  This is a well-structured and relevant study addressing an important occupational health question. The integration of validated behavioral instruments with objective clinical data is a strength.

Minor revisions are suggested:

1. Briefly justify covariate selection in adjusted models or acknowledge potential residual confounding (e.g., occupational differences).

2. Clearly define circadian misalignment in the Methods section.

3. Slightly soften wording suggesting better health among rotating-shift workers, given the cross-sectional design.

4. Confirm statistical assumption checks.

5. Ensure compliance with PLOS data availability requirements.

6. Correct minor typographical errors.

**Reviewer #2:** The manuscript has many strengths. It addresses an important occupational, cardiovascular and public health concern, used validated instruments and standardized clinical measurements, and reported following sound ethical principles. The inclusion of multiple lifestyle and cardiometabolic variables in a a good sample of healthcare worker population is a notable strength and provides useful descriptive insights.

However, a crucial limitation that impacts the most favorable review is that the conclusions seem to overstate the findings based on the current data presented. Of most importance is that after adjustment for confounders, most cardiometabolic outcomes were not significantly associated with rotating-shift work, yet broader claims about better health status were made for shift workers. The cross-sectional design limits causal inference, and the restriction of the study participants to only normoglycemic workers, and in one hospital may reduce generalizability.

The manuscript briefly mentions the healthy worker effect, but this issue likely plays an important role in the observed differences between groups. The shift workers shifts may represent a healthier subset of employees, while unhealthier individuals may transition to the day shift. This could partially explain the more favorable lifestyle, particularly in physical activity, the only variable that remained statistically significant. This possibility should be discussed more explicitly

The manuscript would benefit from substantially revising the conclusions to align with the adjusted results. This will improve clarity and transparency of the statistical analyses, and more fully address confounding and study limitations.

**Reviewer #3:** This publication examines a significant and relevant issue in occupational health: the correlation between rotating-shift work and cardiometabolic and lifestyle outcomes among hospital personnel. The amalgamation of clinical indicators with established behavioural and sleep-related instruments is praiseworthy and enhances the study's usefulness.

However, the manuscript must address some significant methodological and interpretation concerns before it can be considered for publication.

Principal Observations

1. Research methodology and causal inference

The cross-sectional design fundamentally restricts causal inference. The text often suggests that rotating-shift work has protective benefits (e.g., healthier lifestyle, reduced cardiovascular risk), a claim not supported by the study design. These interpretations should be amended to indicate association solely, and the potential for reverse causality must be expressly addressed.

2. Healthy worker effect and selection bias

A significant issue is the probability of a pronounced healthy worker effect. The rotating-shift cohort is markedly younger and has a distinct vocational makeup compared with day workers. These discrepancies are likely to skew the results in favour of a more advantageous health profile among shift workers.

The existing adjustment technique (age, sex, BMI, physical activity) is inadequate to completely mitigate this bias. The authors ought to contemplate:

Stratified analyses (e.g., by age or professional category), or

More sophisticated adjustment methodologies (e.g., propensity score techniques)

This limitation must be significantly broadened in the discourse.

3. Remaining confounding factors

Significant confounders were insufficiently addressed, including:

Duration and cumulative exposure to nocturnal work shifts

Socioeconomic status

Workload and occupational stress

Comprehensive sleep scheduling and circadian disturbance

These characteristics are very pertinent to shift work studies and may considerably affect the results.

Definition of exposure

The definition of "rotating-shift work" is inadequately detailed. The characterisation is ambiguous:

Frequency and intensity of nocturnal shifts

Length of exposure (years)

Rotational patterns

The absence of this information constrains the interpretation of the findings. If data are accessible, dose–response analyses ought to be contemplated.

5. Analysis of Results

The observation that rotating-shift workers display superior cardiometabolic profiles contradicts extensive epidemiological research. This gap is probably attributable to prejudice (e.g., selection bias, residual confounding) rather than an actual protective effect.

The discourse requires revision to:

Refrain from excessive interpretation

Conduct a more rigorous comparison with current meta-analyses.

Highlight alternate interpretations (e.g., behavioural compensation, selection biases)

6. Statistical Examination

The application of GLM is suitable; nonetheless, the statistical analysis is deficient in detail and rigour.

The model assumptions are not disclosed.

No interaction analyses, such as sex-by-shift work, are provided.

No sensitivity analyses were performed.

These issues should be resolved to enhance the validity of the findings.

7. Sample Size and Statistical Power

The limited sample size (n = 225) may constrain statistical power, especially for subgroup analysis. A rationale for the sample size or a declaration of reduced statistical power is advisable.

8. Accessibility of Data

The manuscript indicates that data are accessible "upon request", which contravenes PLOS data sharing policy. The authors must either upload the dataset to a public repository or provide a clear justification for any limits imposed.

Marginal Observations

Minor typographical problems exist (e.g., repetition of “rotating rotating-shift”).

Tables would be enhanced by incorporating effect sizes alongside p-values.

The abstract should avoid causal language and more accurately reflect the cross-sectional nature of the study.

Recent literature from 2023 to 2025 should be integrated to enhance the discourse.

Final Assessment

This manuscript possesses potential and tackles a pertinent public health concern. Significant modifications are necessary, especially regarding methodological rigour, bias control, and the interpretation of findings. I suggest a substantial change prior to additional evaluation.

**Reviewer #4:**  Generally, the manuscript is well articulated and meets the standards for publication.

It provides insights on an emerging issue that needs to be addressed by policy makers in the health sector.

The Authors could however consider the following comments:

1. Line 88, delete the word rotating and remain with rotating-shift.

2. Line 99, instead of using the term discrepant, use the word different/contrary.

3. Line 113, I suggest including the name of the institution and not using the term single institution in Spain.

4. In the study design, explain how the study size was arrived at. What sampling technique was used and the assumptions.

5. Line 151, amend to were also assessed during this examination.

6. In the tables 1.1 and 1.2, I suggest to add the N(%) across the columns.

7. I have concerns on the p-values reported and the tests used in Tables 1.1 and 1.2:

A one-way ANOVA is used to determine if there are statistically significant differences between the means of three or more independent (unrelated) groups. It evaluates the impact of a single factor—a categorical independent variable—on a continuous dependent variable.

I recommend to use independent t-test to compare the groups.

Also, for the p-values reported for Job Category, it would be inaccurate to report the chi-square for that since some of the cell frequencies are less than 5. I suggest to use the Fishers exact test.

And also for Cardiovascular Risk, some of the cells are less than 5. Therefore, chi-square will not be accurate for the p-value estimate.

8. Line 196, can we really talk of more likely for an association test? Likelihoods are estimated using a regression analysis. I suggest you say "rotating-shift workers were majority women".

9. Line 328, I suggest use the word differences.

10. On the conclusion, what can policy makers deduce from this study? A statement from the researchers can come along away.

You could think around:

i. Shift to Health Promotion over Risk Avoidance: Instead of just limiting hours, policy should incentivize programs that encourage physical activity and sleep hygiene, as these specific factors appear to "enhance the resilience" of the workforce.

ii. Context is a Lever for Intervention: Since individual and contextual factors mitigate risk, policy should focus on workplace environments (e.g., providing healthy food options, gym access, or optimized lighting for circadian adaptation) to build worker resilience.

**********

6. PLOS authors have the option to publish the peer review history of their article (what does this mean?). If published, this will include your full peer review and any attached files.

**Do you want your identity to be public for this peer review?**  If you choose “no”, your identity will remain anonymous but your review may still be made public.

For information about this choice, including consent withdrawal, please see our Privacy Policy.

Reviewer #1: No

Reviewer #2: No

Reviewer #3: No

Reviewer #4: **Yes:** Randy Ogana

**********

[NOTE: If reviewer comments were submitted as an attachment file, they will be attached to this email and accessible via the submission site. Please log into your account, locate the manuscript record, and check for the action link "View Attachments". If this link does not appear, there are no attachment files.]

-->Figure Resubmissions:

While revising your submission, we strongly recommend that you use PLOS’s NAAS tool (https://ngplosjournals.pagemajik.ai/artanalysis) to test your figure files. NAAS can convert your figure files to the TIFF file type and meet basic requirements (such as print size, resolution), or provide you with a report on issues that do not meet our requirements and that NAAS cannot fix.

After uploading your figures to PLOS’s NAAS tool - https://ngplosjournals.pagemajik.ai/artanalysis, NAAS will process the files provided and display the results in the "Uploaded Files" section of the page as the processing is complete. If the uploaded figures meet our requirements (or NAAS is able to fix the files to meet our requirements), the figure will be marked as "fixed" above. If NAAS is unable to fix the files, a red "failed" label will appear above. When NAAS has confirmed that the figure files meet our requirements, please download the file via the download option, and include these NAAS processed figure files when submitting your revised manuscript.

# Author comment: Author response to Decision Letter 1

**DOI:** 10.1371/journal.pgph.0006956.r002

16 Apr 2026

Attachment Submitted filename: Response to reviewers.docx

# Peer review: Decision Letter 1

**DOI:** 10.1371/journal.pgph.0006956.r003

**Authors:** Sarah Jose

28 May 2026

PGPH-D-25-03895R1

Relationship of rotating-shift work with lifestyle and health status of hospital employees

PLOS Global Public Health

Dear Dr. Galmes-Panades,

Thank you for submitting your manuscript to PLOS Global Public Health. After careful consideration, we feel that it has merit but does not fully meet PLOS Global Public Health’s publication criteria as it currently stands. Therefore, we invite you to submit a revised version of the manuscript that addresses the points raised during the review process.

Please review the feedback from the reviewers and address the points raised by reviewer 4.

Please submit your revised manuscript by Jun 27 2026 11:59PM. If you will need more time than this to complete your revisions, please reply to this message or contact the journal office at globalpubhealth@plos.org. When you're ready to submit your revision, log on to https://www.editorialmanager.com/pgph/ and select the 'Submissions Needing Revision' folder to locate your manuscript file.

Please include the following items when submitting your revised manuscript:

- A letter that responds to each point raised by the editor and reviewer(s). You should upload this letter as a separate file labeled 'Response to Reviewers'.
- A marked-up copy of your manuscript that highlights changes made to the original version. You should upload this as a separate file labeled 'Revised Manuscript with Track Changes'.
- An unmarked version of your revised paper without tracked changes. You should upload this as a separate file labeled 'Manuscript'.

Guidelines for resubmitting your figure files are available below the reviewer comments at the end of this letter.

As the corresponding author, your ORCID iD is verified in the submission system and will appear in the published article. PLOS supports the use of ORCID, and we encourage all coauthors to register for an ORCID iD and use it as well. Please encourage your coauthors to verify their ORCID iD within the submission system before final acceptance, as unverified ORCID iDs will not appear in the published article. *Only*  the individual author can complete the verification step; PLOS staff *cannot*  verify ORCID iDs on behalf of authors.

We look forward to receiving your revised manuscript.

Kind regards,

Sarah Jose, Ph.D.

Staff Editor

PLOS Global Public Health

Journal Requirements:

If the reviewer comments include a recommendation to cite specific previously published works, please review and evaluate these publications to determine whether they are relevant and should be cited. There is no requirement to cite these works unless the editor has indicated otherwise.

Please review your reference list to ensure that it is complete and correct. If you have cited papers that have been retracted, please include the rationale for doing so in the manuscript text, or remove these references and replace them with relevant current references. Any changes to the reference list should be mentioned in the rebuttal letter that accompanies your revised manuscript. If you need to cite a retracted article, indicate the article’s retracted status in the References list and also include a citation and full reference for the retraction notice.

Additional Editor Comments (if provided):

[Note: HTML markup is below. Please do not edit.]

Reviewers' comments:

Reviewer's Responses to Questions

**Comments to the Author**

1. If the authors have adequately addressed your comments raised in a previous round of review and you feel that this manuscript is now acceptable for publication, you may indicate that here to bypass the “Comments to the Author” section, enter your conflict of interest statement in the “Confidential to Editor” section, and submit your "Accept" recommendation.

Reviewer #2: All comments have been addressed

Reviewer #4: (No Response)

**********

2. Does this manuscript meet PLOS Global Public Health’s publication criteria? Is the manuscript technically sound, and do the data support the conclusions? The manuscript must describe methodologically and ethically rigorous research with conclusions that are appropriately drawn based on the data presented.

Reviewer #2: Yes

Reviewer #4: Yes

**********

3. Has the statistical analysis been performed appropriately and rigorously?

Reviewer #2: Yes

Reviewer #4: Yes

**********

4. Have the authors made all data underlying the findings in their manuscript fully available (please refer to the Data Availability Statement at the start of the manuscript PDF file)?

The PLOS Data policy requires authors to make all data underlying the findings described in their manuscript fully available without restriction, with rare exception. The data should be provided as part of the manuscript or its supporting information, or deposited to a public repository. For example, in addition to summary statistics, the data points behind means, medians and variance measures should be available. If there are restrictions on publicly sharing data—e.g. participant privacy or use of data from a third party—those must be specified.

Reviewer #2: Yes

Reviewer #4: Yes

**********

5. Is the manuscript presented in an intelligible fashion and written in standard English?

PLOS Global Public Health does not copyedit accepted manuscripts, so the language in submitted articles must be clear, correct, and unambiguous. Any typographical or grammatical errors should be corrected at revision, so please note any specific errors here.

Reviewer #2: Yes

Reviewer #4: Yes

**********

6. Review Comments to the Author

Please use the space provided to explain your answers to the questions above. You may also include additional comments for the author, including concerns about dual publication, research ethics, or publication ethics. (Please upload your review as an attachment if it exceeds 20,000 characters)

Reviewer #2: Dear Authors,

You have done an excellent job with addressing the reviewer concerns/comments. Your point by point response to all comments is commendable. Keep up the good work.

Reviewer #4: Thanks to the authors for their extensive changes in the manuscript. There has been a significant improvement in the new version in terms of methodological clarity, statistical strength, interpretation of findings and overall presentation.

In particular, the authors have:

1. Clarified the exposure definition and rotating-shift schedule,

2. Revised the statistical analyses appropriately (including replacement of ANOVA with independent t-tests where applicable and use of Fisher’s exact test when assumptions were violated)

3. Clarified model assumptions and interaction analyses,

4. strengthened the interpretation of findings and policy implications, and complied with PLOS data availability requirements through deposition in Dryad.

5. The revised conclusions are now substantially more cautious and appropriately framed for a cross-sectional study.

I only have a minor remaining concern regarding the methodology. The authors have noted that they did not pre-calculate the sample size, but they should make this clear and be consistent across the manuscript in the Method section, indicating that the analyses they present were based on a post hoc power/detectable effect estimate and not a pre-calculated sample size.

Additionally, they state that the sample was 240, but what is reported in the tables is 225. I suggest you include the response rate in the results section. As it stands, it raises questions about whether the 15 excluded participants introduce selection bias and whether the study remains adequately powered at n=225 rather than n=240.

Overall, the manuscript, as a whole, has been greatly improved and is now scientifically acceptable. If the description of the sample size is clarified, it should suffice.

**********

7. PLOS authors have the option to publish the peer review history of their article (what does this mean?). If published, this will include your full peer review and any attached files.

**Do you want your identity to be public for this peer review?**  If you choose “no”, your identity will remain anonymous but your review may still be made public.

For information about this choice, including consent withdrawal, please see our Privacy Policy.

Reviewer #2: No

Reviewer #4: No

**********

[NOTE: If reviewer comments were submitted as an attachment file, they will be attached to this email and accessible via the submission site. Please log into your account, locate the manuscript record, and check for the action link "View Attachments". If this link does not appear, there are no attachment files.]

Figure Resubmissions:

While revising your submission, we strongly recommend that you use PLOS’s NAAS tool (https://ngplosjournals.pagemajik.ai/artanalysis) to test your figure files. NAAS can convert your figure files to the TIFF file type and meet basic requirements (such as print size, resolution), or provide you with a report on issues that do not meet our requirements and that NAAS cannot fix.

After uploading your figures to PLOS’s NAAS tool - https://ngplosjournals.pagemajik.ai/artanalysis, NAAS will process the files provided and display the results in the "Uploaded Files" section of the page as the processing is complete. If the uploaded figures meet our requirements (or NAAS is able to fix the files to meet our requirements), the figure will be marked as "fixed" above. If NAAS is unable to fix the files, a red "failed" label will appear above. When NAAS has confirmed that the figure files meet our requirements, please download the file via the download option, and include these NAAS processed figure files when submitting your revised manuscript.

# Author comment: Author response to Decision Letter 2

**DOI:** 10.1371/journal.pgph.0006956.r004

2 Jun 2026

Attachment Submitted filename: Response to reviewers_June 2026.docx

# Peer review: Decision Letter 2

**DOI:** 10.1371/journal.pgph.0006956.r005

**Authors:** Julia Robinson

12 Jul 2026

Relationship of rotating-shift work with lifestyle and health status of hospital employees

PGPH-D-25-03895R2

Dear PhD Galmes-Panades,

We are pleased to inform you that your manuscript 'Relationship of rotating-shift work with lifestyle and health status of hospital employees' has been provisionally accepted for publication in PLOS Global Public Health.

Before your manuscript can be formally accepted you will need to complete some formatting changes, which you will receive in a follow up email. A member of our team will be in touch with a set of requests.

Please note that your manuscript will not be scheduled for publication until you have made the required changes, so a swift response is appreciated.

IMPORTANT: The editorial review process is now complete. PLOS will only permit corrections to spelling, formatting or significant scientific errors from this point onwards. Requests for major changes, or any which affect the scientific understanding of your work, will cause delays to the publication date of your manuscript.

If your institution or institutions have a press office, please notify them about your upcoming paper to help maximize its impact. If they'll be preparing press materials, please inform our press team as soon as possible -- no later than 48 hours after receiving the formal acceptance. Your manuscript will remain under strict press embargo until 2 pm Eastern Time on the date of publication. For more information, please contact globalpubhealth@plos.org.

Thank you again for supporting Open Access publishing; we are looking forward to publishing your work in PLOS Global Public Health.

Best regards,

Julia Robinson

Executive Editor

PLOS Global Public Health

***********************************************************

Reviewer Comments (if any, and for reference):

Reviewer's Responses to Questions

**Comments to the Author**

1. If the authors have adequately addressed your comments raised in a previous round of review and you feel that this manuscript is now acceptable for publication, you may indicate that here to bypass the “Comments to the Author” section, enter your conflict of interest statement in the “Confidential to Editor” section, and submit your "Accept" recommendation.

Reviewer #2: All comments have been addressed

**********

2. Does this manuscript meet PLOS Global Public Health’s publication criteria? Is the manuscript technically sound, and do the data support the conclusions? The manuscript must describe methodologically and ethically rigorous research with conclusions that are appropriately drawn based on the data presented.

Reviewer #2: Yes

**********

3. Has the statistical analysis been performed appropriately and rigorously?

Reviewer #2: Yes

**********

4. Have the authors made all data underlying the findings in their manuscript fully available (please refer to the Data Availability Statement at the start of the manuscript PDF file)?

The PLOS Data policy requires authors to make all data underlying the findings described in their manuscript fully available without restriction, with rare exception. The data should be provided as part of the manuscript or its supporting information, or deposited to a public repository. For example, in addition to summary statistics, the data points behind means, medians and variance measures should be available. If there are restrictions on publicly sharing data—e.g. participant privacy or use of data from a third party—those must be specified.

Reviewer #2: Yes

**********

5. Is the manuscript presented in an intelligible fashion and written in standard English?

PLOS Global Public Health does not copyedit accepted manuscripts, so the language in submitted articles must be clear, correct, and unambiguous. Any typographical or grammatical errors should be corrected at revision, so please note any specific errors here.

Reviewer #2: Yes

**********

6. Review Comments to the Author

Please use the space provided to explain your answers to the questions above. You may also include additional comments for the author, including concerns about dual publication, research ethics, or publication ethics. (Please upload your review as an attachment if it exceeds 20,000 characters)

Reviewer #2: (No Response)

**********

7. PLOS authors have the option to publish the peer review history of their article (what does this mean?). If published, this will include your full peer review and any attached files.

**Do you want your identity to be public for this peer review?**  If you choose “no”, your identity will remain anonymous but your review may still be made public.

For information about this choice, including consent withdrawal, please see our Privacy Policy.

Reviewer #2: No

**********
