# Supplementary material for: Correction: Relationship of rotating-shift work with lifestyle and health status of hospital employees
Source: PLOS Glob Public Health. 2026 Sep 29;6(9):e0007404. doi: 10.1371/journal.pgph.0007404 (PMC13623138; doi:10.1371/journal.pgph.0007404)
Supplement: Supplementary file 1 [file pgph.0007404.s001.docx]

**S1 Table** Bivariate analysis of the association of different variables with sex

| **Variable** | **All**  **(n=225)** | **Women**  **(n=176)** | **Men**  **(n=49)** | **p-value** |
| --- | --- | --- | --- | --- |
| Mean ± SD | | | | |
| ***Clinical variables*** | | | | |
| Glucose (mg/dL) | 88.8 ± 12.2 | 89.0 ± 12.9 | 88.4 ± 9.5 | 0.764 |
| Total Cholesterol (mg/dL) | 187.4 ± 36.6 | 184.9 ± 35.6 | 196.3 ± 39.1 | 0.054 |
| HDL Cholesterol (mg/dL) | 59.4 ±13.3 | 61.8 ± 13.2 | 50.9 ±10.1 | <0.001* |
| LDL Cholesterol (mg/dL) | 113.5 ± 32.2 | 110.1 ± 29.9 | 125.6 ± 37.0 | 0.003* |
| Triglycerides (mg/dL) | 77.7 ± 59.9 | 71.5 ± 34.3 | 99.9 ± 59.9 | <0.001* |
| Systolic Blood Pressure (mmHg) | 122.2 ± 14.8 | 120.1 ± 14.1 | 129.9 ± 14.9 | <0.001* |
| Diastolic Blood Pressure (mmHg) | 75.4 ± 9.6 | 74.4 ± 9.1 | 78.9 ± 10.7 | 0.004* |
| ***Anthropometric variables*** |  |  |  |  |
| BMI (kg/m²) | 24.5 ± 5.0 | 24.1 ± 5.1 | 26.0 ± 4.4 | 0.018* |
| Waist Circumference (cm) | 79.8 ± 12.6 | 77.0 ± 11.5 | 89.6 ±11.7 | <0.001* |
| ***Sociodemographic and occupational variables*** | | | | |
| Age (years) | 42.2 ± 11.2 | 42.9 ± 11.3 | 39.9 ± 10.4 | 0.093 |
| Type of shift work (years) | 12.5 ± 9.7 | 12.5 ± 9.8 | 9.7 ± 9.1 | 0.078 |
| Living with others  No  Yes | 57 (25.3)  168 (74.7) | 42 (23.9)  134 (76.1) | 15 (30.6)  34 (69.4) | 0.337 |
| Marital Status  In a relationship  Single | 135 (60.0)  90 (40.0) | 105 (59.7)  71 (40.3) | 30 (61.2)  19 (38.8) | 0.843 |
| Education Level  University Degree  Non-University Degree | 140 (62.2)  85 (37.8) | 111 (63.1)  65 (36.9) | 29 (59.2)  20 (40.8) | 0.620 |
| Job Category  Nursing Staff, Midwives and Residents  Physicians and Medical Residents  Orderlies  Others | 110 (48.9)  43 (19.1)  17 (7.6)  55 (24.4) | 93 (52.8)  27 (15.3)  8 (4.6)  48 (27.3) | 17 (34.7)  16 (32.7)  9 (18.4)  7 (14.3) | <0.001* |
| Contract Type  Permanent  Resident  Temporary | 144 (64.0)  44 (19.6)  37 (16.4) | 116 (65.9)  32 (18.2)  28 (15.9) | 28 (57.1)  12 (24.5)  9 (18.4) | 0.501 |
| Morning Shift Duration  7–10 h  12–24 h | 194 (86.2)  31 (13.8) | 147 (83.5)  29 (16.5) | 47 (95.9)  2 (4.1) | 0.026* |
| Night-Shift Duration  7–10 h  12–24 h | 59 (47.2)  66 (52.8) | 41 (44.6)  51 (55.4) | 18 (54.6)  15 (45.5) | 0.324 |
| ***Lifestyle variables*** | | | | |
| Adherence to Mediterranean Diet | 8.7 ± 1.9 | 8.8 ± 1.9 | 8.4 ± 1.8 | 0.185 |
| Light Physical Activity (min/day) | 1.4 ± 6.0 | 1.7 ± 6.6 | 0.6 ± 3.0 | 0.281 |
| Moderate Physical Activity (min/day) | 44.1 ± 63.6 | 42.6 ± 65.8 | 49.4 ± 55.3 | 0.512 |
| Vigorous Physical Activity (min/day) | 20.3 ± 40.9 | 13.6 ± 28.6 | 44.2 ± 63.7 | <0.001* |
| Total Physical Activity (min/day) | 65.8 ± 81.3 | 57.8 ± 77.7 | 94.2 ± 88.5 | 0.005* |
| Smoke  Non-smoker  Smoker  Former Smoker | 139 (61.8)  35 (15.6)  51 (22.67) | 107 (60.8)  27 (15.3)  42 (23.9) | 32 (65.31)  8 (16.3)  9 (18.4) | 0.719 |
| ***Sleep factors variables*** | | | | |
| Night Shifts per Month  ≤4  >4 | 137 (60.9)  88 (39.1) | 112 (63.6)  64 (36.4) | 25 (51.0)  24 (49.0) | 0.109 |
| Sleep Quality  Good  Bad | 101 (44.9)  124 (55.1) | 74 (42.1)  102 (58.0) | 27 (55.1)  22 (44.9) | 0.104 |
| Sleep Duration  ≥7 h  <7 h | 52 (23.1)  173 (76.9) | 44 (25.0)  132 (75.0) | 8 (16.3)  41 (83.7) | 0.203 |
| Sleep Medication  No  Yes | 182 (80.9)  43 (19.1) | 139 (79.0)  37 (21.0) | 43 (87.8)  6 (12.2) | 0.167 |

*Abbreviations*: HDL: High-Density Lipoprotein; LDL: Low-Density Lipoprotein; BMI: Body Mass Index.

An asterisk indicates a significant difference (p < 0.05) between groups.

^a^ Values are indicated as mean ± SD for continuous variables and n (%) for categorical variables. Independent t-test and χ^2^ tests were used to compare continuous and categorical variables, respectively.

^b^ The ‘Others’ category includes administrative assistant, administrative staff, social worker, psychologist, clinical psychology resident, pharmacist, pathology technician, biologist, telephone operator, management technician, and X-ray technician.
